# Height-for-age and weight-for-age growth charts for Pakistani infants under six months: derived from a novel case selection method using multiple indicator cluster survey data

**DOI:** 10.1186/s12874-023-02116-y

**Published:** 2023-12-08

**Authors:** Muhammad Aasim, Sohail Chand

**Affiliations:** 1 NHRC, NIH (HRI) Research Centre, Shaikh Zayed Medical Complex, Lahore, Pakistan; 2 College of Statistical Sciences, University of the Punjab, Lahore, Pakistan

**Keywords:** Breastfed, Child growth standards, MICS data, Novel case selection method, Pakistan

## Abstract

**Background:**

In the past two decades, there has been a growing recognition of the need to establish indigenous standards or reference growth charts, particularly following the WHO multicenter growth study in 2006. The availability of accurate and reliable growth charts is crucial for monitoring child health. The choice of an appropriate model for constructing growth charts depends on various data characteristics, including the distribution’s tails and peak. While Pakistan has reported some reference growth charts, there is a notable absence of indigenous charts for children under two years of age, especially for infants aged 0–6 months who are exclusively breastfed. Additionally, acquiring data poses a significant challenge, particularly for low-income countries, as it demands substantial resources such as finances, time, and expertise. The Multiple Indicator Cluster Survey (MICS) constitutes a large-scale national survey conducted periodically in low-income countries under the auspices of UNICEF. In this study, we propose methods for generating selection variables utilizing the “Novel Case Selection Method,“ as previously published. Further our approach enables to select and fit appropriate model to the MICS data, selected, and to develop the standard growth charts.

**Methods:**

Out of the 11,478 children under 6 months of age included in MICS-6 (Pakistan), 3,655 children (1,831 males and 1,824 females) met the specified criteria and were selected using the “Novel Case Selection Method”. The sample was distributed across provinces as follows: 841 (23.0%) from KPK, 1,464 (40.1%) from Punjab, 819 (22.4%) from Sindh, and 531 (14.5%) from Balochistan. This sample encompassed both rural (76.4%) and urban (23.6%) populations. Following data cleaning and outlier removal, a total of 3,540 records for weight (1,768 males and 1,772 females) and 3,515 records for height (1,759 males and 1,756 females) were ultimately available for the development of standard charts. The Bayesian Information Criterion (BIC) was employed to determine the optimal degrees of freedom for L, M, and S using *RefCurv_0.4.2*. Three families within the gamlss class—namely, Box Cox Cole and Green (BCCG), Box Cox T (BCT), and Box Cox Power Exponential (BCPE)—were applied, each with three smoothing techniques: penalized splines (ps), cubic splines (cs), and polynomial splines (poly). The best-fitted model was selected from these nine combinations based on the Akaike Information Criteria.

**Results:**

The Novel Case Selection Method yielded 3655 cases as per criteria. After cleaning the data, this method lead to selection of 3540 children for “weight for age” (W/A) and 3515 children for “height for age” (H/A). The “BCPE” family and “ps” as smoothing method proved to be best on AIC for all four curves, i.e. the W/A male, W/A female, H/A male, and H/A female. The optimum selected degrees of freedom for the curve “W/A”, for both genders were (M = 1, L = 0, S = 0). The optimum degrees of freedom for H/A male were again (M = 1, L = 0, S = 0), but for females the selected degrees of freedom were (M = 1, L = 1, S = 1). The indigenous fitted standard curves for Pakistan were on lower trajectory in comparison to WHO standards.

**Conclusion:**

This study uses the Novel Case Selection Method with introduced algorithms to construct tailored growth charts for lower and middle-income countries. Leveraging extensive MICS data, the methodology ensures representative national samples. The resulting charts hold practical value and await validation from established data sources, offering valuable tools for policy makers and clinicians in diverse global contexts.

**Supplementary Information:**

The online version contains supplementary material available at 10.1186/s12874-023-02116-y.

## Background

Height for age (H/A) and weight for age (W/A) are pivotal measures for assessing children’s growth against standard patterns [1]. Presently, Pakistan relies on WHO standards for monitoring child growth and estimating rates of wasting and stunting in the population [2]. Over the past decade, several studies have been conducted in Pakistan to evaluate and monitor child growth. For instance, one study examined the nutritional status of school children aged 5 to 12 years, reporting the prevalence of overweight and obesity based on the WHO 2007 standards [3]. Another study by Aziz et al. in 2012 encompassed a national sample of 12,837 children aged 3 to 16 years, developing percentile charts for children aged 5 to 14. Notably, this study compared its percentiles with CDC references rather than WHO [4]. Subsequently, in 2018, a study established reference growth charts for Pakistani children aged 4 to 15 years, drawing from 9,515 students across four cities: Karachi, Larkana, Quetta, and Peshawar. The primary objective was to study tooth eruption among healthy children [5]. The most recent study, conducted by Asif et al. in 2022, was based on cross-sectional data from 10,782 children aged 2 to 19 years, gathered through a multiethnic anthropometric survey conducted in 2016. Among these, 10,668 were included to develop H/A z-scores for boys and girls. Notably, this study compared the median height (z = 0) with WHO and other international studies. Interestingly, it stands as the first Pakistani study to report a median height above WHO standards until the age of 8 years. From ages 9 to 18, the median height was lower than that of WHO [6]. Additionally, another study was observed, which assessed the centiles for BMI, height-for-age, and weight-for-age. It reported that their BMI centiles were higher than the WHO centiles up to the age of 8 years, and lower during the pubertal period. Specifically, the BMI and height-for-age centiles were lower between ages 8 and 10 years [7]. However, none of these studies encompassed children under two years of age, especially those who were exclusively breastfed, highlighting a significant research gap.

It is almost established now that each country needs its own indigenous standards of growths [8–11], however the development of charts is not a simple and straightforward job for many reasons. Proper representation of national sample, selection of cases, and selection of model are few of the challenges to meet and need lot of care, discussed in detail as under.

For developing the standards, the most important condition imposed for including children of age less than 6 months is their exclusive breast feeding. Also the child must be singleton, full term, with birth order up to 4, having no severe illness episode and must born to a non-smoking mother [1]. Preparation of data with fulfilling the given conditions and also having national representation is an uphill task. It was suggested by Khan et al. 2019, that the multiple indicator cluster survey (MICS) has a robust data set on children and women across the board [12]. The Novel Case Selection Method [13] was suggested earlier for children of age 0 to 24 months, based on round 6, after looking into MICS data of round 4, 5 and 6. The data for the round 4 and 5 were discarded after having a detailed data view [13].

Many of the studies used the cross-sectional datasets collected for other purposes to develop the growth charts [11, 14, 15]. This MICS data also collects the anthropometry measures of children for 0–5 years age. Though these data are collected for the purpose of wasting and stunting estimation at national level, but many such useful information are available which can help selection for development of standards. Some of the variables are directly available while other variables can be generated using the information available in the dataset.

Once the dataset is made available, there are many methods available in literature which can be used for development of growth charts. The WHO working group [16] studied 30 different methods [17] and recommended Generalized Additive Models for Location, Scale and Shape (GAMLSS) [18]. This was the generalization of “LMS” method by Cole and Green [19] based on box-cox transformation which is considered the ultimate transformation of data into normal distribution. WHO multicenter growth study [16] ultimately used Box-Cox Power Exponential (BCPE) method to develop the standard charts for 0–5 years [16]. This method was modification of “LMS” and was suggested by Rigby and Stasinopoulos for the data which required adjustments for abnormal kurtosis [20]. Another method under GAMLSS is the Box-Cox T (BCT) [21], which makes adjustment for the heavy tails.

Smoothing of curves is also important aspect for development of growth charts. It helps not only in prediction between any two “x” values by interpolation but also helps to extrapolate. For GAMLSS class models well worked out smoothing techniques are penalized splines (ps), cubic splines (cs) and polynomials (poly) [22]. There are other smoothing techniques like LOWESS, quantile, etc. but are not under consideration in this paper for reason of parsimonious modeling.

This study mainly focuses on the addition of algorithm details in “Novel Case Selection Method” [13] based on MICS data, making final selection of cases and development of Pakistani standards for exclusively breastfed children of age 0–6 months. The development is performed by comparing three families of GAMLSS class, i.e. BCCG, BCPE and BCT in combination with three smoothing techniques (ps), (cs) and (poly). Finally the developed indigenous charts are compared with WHO standards.

Objectives: The primary goal of this study is to establish growth standards specific to exclusively breastfed Pakistani children aged 0 to 6 months. This is achieved by employing the Novel Case Selection Method [13] on data obtained from MICS. Subsequently, we assessed and compared the performance of three GAMLSS families (BCCG, BCPE, BCT) in conjunction with different smoothing techniques (ps, cs, poly) to select the model. Finally, we conducted a comprehensive comparison of the standard developed through final fitted model with the existing WHO standards.

## Methods

This section comprises of three major components. One is the selection of children from MICS-6 data file through, “Novel Case Selection Method” [13] and the second is the selection of optimum model and fitting. While the third component is the comparison of developed indigenous and WHO standards. Before dealing these three components, the characteristics of MICS-6 data are elaborated here first:

### Characteristics of MICS-6 data

The MICS is a regularly conducted survey in Pakistan, utilizing a two-stage sample design. It is administered by each province and ensures high-quality data due to its comprehensive sample design, well-trained data collection staff, and robust monitoring system. Currently, this survey is conducted in 116 countries worldwide, with technical support from UNICEF, and the datasets are accessible on their website. In every district, both rural and urban areas are identified as strata. First, census enumeration areas are selected using systematic random sampling with probability proportional to size, followed by the selection of clusters of 20 households from each enumeration area using systematic random sampling. This method creates a representative sample from each province. Moreover, the data is collected in 11 different modules, each of which is stored in a separate data file in .sav format. For our study, we focused on the household file (hh), birth history file (bh), children file (ch), and women file (wm). These files were analyzed, and the main framework for the “Novel Case Selection Method“ [13] was formulated, as outlined in our previous study [13]. All children under 5 years of age had their weights and heights measured using anthropometric equipment recommended by UNICEF [23]. Height was recorded in centimeters, weight in kilograms, and age in days, months, and years, along with the gender of each child.

### Inclusion and exclusion criteria

Children under six months of age were selected based on specific criteria, including being singletons, born at full term, belonging to birth order up to four and exclusively breastfed. Those born to non-smoking mothers, having severe illnesses, and residing in congested households were excluded.

### Novel case selection method

The “Novel Case Selection Method“ [13] employed in this study represents a systematic approach to identifying and refining crucial variables for the development of growth standards. This method involves creating new variables, if needed, and judiciously utilizing existing ones to meet the stringent criteria set forth by the World Health Organization (WHO). Specifically, for children to meet the inclusion and exclusion criteria as mentioned. This innovative approach builds upon a method proposed earlier [13], incorporating slight modifications to further enhance its efficacy. By ensuring that the selected variables strictly adhere to WHO guidelines, this method lays a solid foundation for subsequent analyses and the creation of accurate growth charts.

The data for Punjab, Sindh, KPK, and Balochistan from MICS round 6 was acquired from the official UNICEF website, and a master file containing all essential variables was meticulously compiled. Notably, three pivotal variables—exclusive breastfeeding, absence of severe illnesses, and non-congested household living—were not readily accessible in the dataset. To address this, specialized algorithms were devised to derive accurate values for these variables, ensuring comprehensive and precise data for subsequent analyses.

### Exclusive breast fed (EBF)

Initially, two new variables were created: “liquid items” labeled as $X$ and “food items” labeled as  *Y*﻿. The variable as per their name in MICS-6 data file and the names designated for this algorithm process are detailed in table S1 and S2:

There were nine variables containing information about liquids consumed by children in last 24 hours and were designated as ${x}_{1}, {x}_{2}, {\dots x}_{9}$, the detail can be viewed in Table S1.

All were converted to binary variables ${x}_{1}, {x}_{2}, {\dots x}_{9}$(1: Consumed; 0: Not consumed)

The assessment was made by summing up the all nine binary variables as:

$$X=\sum _{i=1}^{9}{x}_{i}$$

Here the variable $X$ presenting the number of liquids consumed in last 24 hours.

Similarly there were 15 variables in MICS-6 file containing information about the solids and semi solids consumed by the children in last 24 hours and they were designated as ${ y}_{1}, {y}_{2}, {\dots y}_{15}$. There detail is available in Table S2.

All were converted into binary variables ${y}_{1}, {y}_{2}, {\dots y}_{15}$(1: Consumed; 0: Not consumed)

The status of children was made clear by summing up these 15 binary variables as:

$$Y=\sum _{i=1}^{15}{y}_{i}$$

Here this variable $Y$ presents the number of solids or semi solids consumed in last 24 hours.

We also utilized the variable BD3: named $Z$, which indicated whether the child was still being breastfed (1 for “Yes” and 0 for “No” as available in the MICS-6 data). Based on these variables, we calculated the status for exclusive breast feeding: $named\, EBF$ using the following criteria:

$$EBF=[1:\left\{If \left(Z=1 \& X+Y=0\right)\right\};0:otehrwise]$$

Here, 1 representing exclusive breastfeeding status and 0 representing cases where exclusive breastfeeding criteria were not met. Thus, individuals selected for the analysis fulfilled the condition $EBF=1$

### Severe illness

We determined the occurrence of severe illness using an algorithm specifically designed for children aged less than six months. The algorithm considered episodes of diarrhea (CA1), fever (CA14), cough (CA16), and difficulty breathing during illness with cough (CA17) in the past 15 days. These variables were directly available in the MICS-6 data. These were named ${L}_{1}, {L}_{2}, {L}_{3}\, and\, {L}_{4}$ respectively with binary categories 1: Yes and 0: No, and “severe illness”, named $L$ was determined as follows:

$$L=\left[1;if {\{L}_{1}=1 \&\left( {L}_{2}=1\left|{L}_{3}=1\right|{L}_{4}=1\right)\right\}:0:otehrwise]$$

The outcome variable $L$ consisted of two categories: 0 denoting no severe illness and 1 denoting the presence of severe illness. Those selected for analysis were the cases where $L=0$.

### Living in non-congested household

To gauge the household’s living conditions, we employed the concept of household congestion, which acts as a proxy for mothers deprived of a suitable environment and proper nutrition. We established a criterion of “2 or fewer” adults per room designated for sleeping, named $C$. This criterion was determined using available variables: the number of household members (HH48, named ${ C}_{1}$), the number of children under age 5 (HH51: ${C}_{2}$), the number of children aged 5–17 years (HH52: ${C}_{3}$), and the count of rooms used for sleeping (HC3: ${C}_{4}$), all of which were directly extracted from the MICS-6 dataset. Using these variables, we calculated the number of adults $A$ as:

$$A={C}_{1}-{(C}_{2}+{C}_{3})$$

Subsequently computed

$$C=\frac{A}{{C}_{4}}$$

The congested status of the household was determined by the condition $C>2$. Children living in households where $C\le 2$ were classified as “living in non-congested households.”

### Curve estimation

Four standard curves were planned for estimation: two for Weight-for-Age (W/A), one for each gender, and two for Height-for-Age (H/A), also segregated by gender. The curve estimation process began with the initial selection of the optimal parameters for the LMS method, accomplished separately for each curve using *RefCurve_0.4.2*. The selection was based on the Bayesian Information Criteria (BIC), aiming to attain the lowest value. This procedure determined the best degrees of freedom for L, M, and S for all four curves. Subsequently, models with three families - Box-Cox Cole and Green (BCCG), Box-Cox Power Exponential (BCPE), and Box-Cox t (BCT) - were fitted for the selected degrees of freedom.

Here the three models used are as under.

### Box-cox cole and green (BCCG)

$$f(y;\lambda ,\mu ,\sigma ,\tau )=\frac{1}{\sigma }{\left[ {1+\lambda (\frac{{y - \mu }}{\sigma })} \right]^{ - 1/\lambda - 1}}exp\left\{ { - \frac{1}{2}{{\left( {\frac{{y - \mu }}{\sigma }} \right)}^2}} \right\}$$

$y$ is the random variable; $\lambda$ is the shape parameter; $\mu$ is the location parameter.

$\sigma$ is the scale parameter;

### Box-cox power exponential (BCPE)

$$\eqalign{f(y;\lambda ,\mu ,\sigma ,\tau ) & =\frac{\tau }{\sigma }{\left[ {1+\lambda (\frac{{y - \mu }}{\sigma })} \right]^{1/\lambda - 1}} \cr & exp\left\{ { - \frac{\tau }{\lambda }\left[ {1 - {{\left( {1+\lambda \left( {\frac{{y - \mu }}{\sigma }} \right)} \right)}^{1/\lambda }}} \right]} \right\}}$$

$\tau$ is an additional shape parameter specific to BCPE

### Box-cox T

$$f(y;\lambda ,\mu ,\sigma ,\nu ,\tau )=\frac{{\tau \Gamma ((\nu +1)/2)}}{{\Gamma (\nu /2)\sqrt {\pi \nu \sigma } }}{\left[ {1+\frac{{{{(\lambda (y - \mu ))}^2}}}{{\nu {\sigma ^2}}}} \right]^{ - (\nu +1)/2}}$$

$\varGamma$*represents the gamma function*.

$\nu$is the degrees of freedom parameter. Specific to BCT

Each candidate model was fitted by taking penalized spline (ps), cubic spline (cs) and Polynomial spline (poly) as smoothing methods and Akaike information criteria (AIC) was calculated.

Here the.

In the context of GAMLSS, penalized splines are used for smooth functions. The mathematical expression for a penalized spline $f(x)$ can be represented as:

$$f(x)=\sum\limits_{{i=1}}^{k} {{\beta _i}{B_i}(x)+\epsilon }$$

${B}_{i}\left(x\right)$ are the basis functions for the spline; ${\beta }_{i}$ are the coefficients associated with each basis function; $k$ is the number of basis functions; $\epsilon$ represents the error term.

Cubic splines are a specific type of piecewise polynomial function used for smoothing. The mathematical expression for a cubic spline $f(x)$ can be represented as:

$$f(x)=a+b(x - c)+c{(x - d)^2}+e{(x - d)^3}, {\rm here}$$

$a, b, c, d\, and\, e$ are coefficients and $x$ is the independent variable.

Polynomial splines are piecewise polynomials that are connected at specific points, known as knots and its mathematical expression for $f\left(x\right)$ is given as:

$$f(x)=\sum\limits_{{i=1}}^{n} {{\beta _i}{x^{(i - 1)}}}$$

${\beta }_{i}$ are the coefficients associated with each polynomial term; $n$ is the degree of the polynomial.

**Fig. 1:**
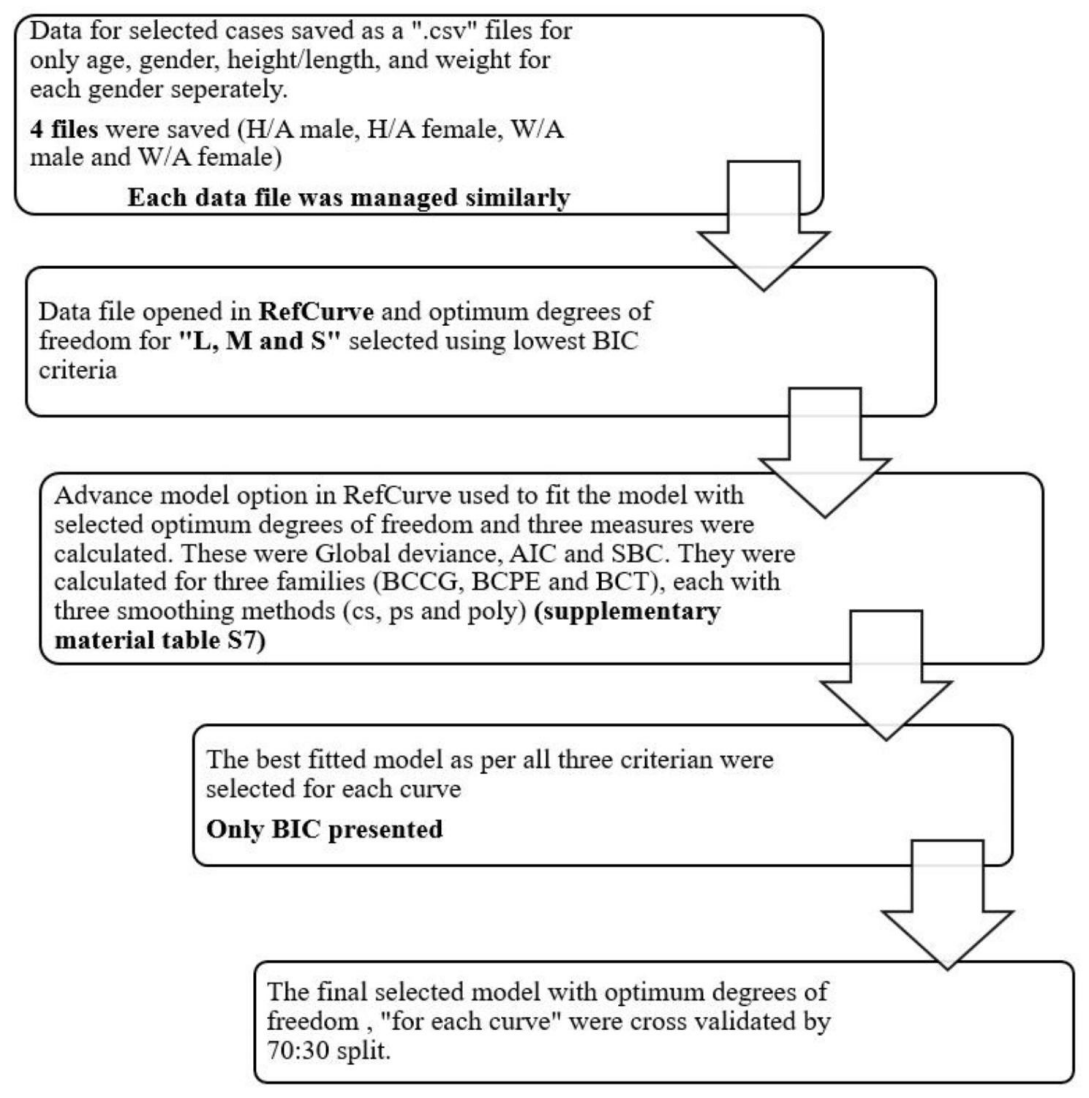
Flowchart illustrating the sequential stages of model selection and validation

The best-fitted model, determined by combining the candidate model with the most suitable smoothing method with the lowest AIC value, was selected for final estimations. These ultimate models underwent cross-validation using a 70:30 split within each data file, with detailed steps outlined in the flow diagram (Fig. 1) and further information provided in the supplementary material (Table S3-S6).

The developed Indigenous Pakistani standards were compared with the WHO multicenter growth standards percentiles for these four selected curves downloaded from their website [23].

## Results

The demographic structure, rural urban and provincial representation by gender of the initially selected 3655 cases is given in (table S8, supplementary material).These selected cases had an overall average age of 12.14 ± 7.36 weeks, with mean weight of 5.00 ± 1.79 kg and height of 56.89 ± 7.79 centimeters (table S8, supplementary material). The eligible children were identified from the MICS dataset utilizing the “novel case selection method“ [13]. Subsequently, the data underwent a rigorous screening process to eliminate any missing or invalid observations, resulting in a final selection of 1768 eligible males for Weight-for-Age (W/A). Among these, the highest count was 97 cases for those aged 2 weeks, while the lowest count was 30 cases for those aged 175 days and above. Similarly, for females, a total of 1772 cases were selected, with the highest count of 104 cases recorded for those at one week of age, and the lowest count of 36 cases for those at 23 weeks. In the case of Height-for-Age (H/A), 1759 males were selected, mirroring the distribution observed for W/A. For females, a total of 1756 cases were selected for H/A. Additionally, descriptive statistics were provided for each week of age (refer to Table 1 for details).

**Table 1:** Cases Selected for Each Age (in Weeks) with Descriptive Measures for Weight and Height, Stratified by Gender

Age (weeks)	Male				Female			
	Weight (kg)		Height (cm)		Weight (kg)		Height (cm)	
	n	Mean ± SD	n	Mean ± SD	n	Mean ± SD	n	Mean ± SD
1	74	3.26 ± 0.82	73	48.74 ± 5.22	104	3.22 ± 0.76	103	48.87 ± 4.85
2	97	3.42 ± 0.84	96	49.53 ± 5.23	99	3.3 ± 0.73	103	49.35 ± 5.63
3	83	3.64 ± 0.71	84	51.35 ± 3.59	92	3.42 ± 0.78	91	50.65 ± 5.37
4	79	3.87 ± 0.81	78	51.65 ± 4.43	82	3.74 ± 0.80	83	51.90 ± 4.43
5	84	3.96 ± 0.93	85	53.22 ± 5.26	68	3.72 ± 0.69	70	53.18 ± 5.69
6	68	3.93 ± 0.87	69	52.84 ± 3.95	76	4.08 ± 0.78	73	53.33 ± 5.03
7	82	4.48 ± 0.87	80	54.76 ± 4.44	73	4.42 ± 1.09	69	54.18 ± 4.49
8	70	4.34 ± 1.04	70	54.53 ± 4.11	75	4.39 ± 0.98	70	55.08 ± 4.18
9	71	4.77 ± 1.02	69	55.98 ± 4.7	75	4.39 ± 0.82	75	54.80 ± 3.82
10	69	5.06 ± 0.85	71	56.36 ± 4.23	71	4.51 ± 1.12	70	55.51 ± 5.20
11	83	4.98 ± 0.93	81	57.52 ± 4.52	71	4.80 ± 0.96	72	56.78 ± 4.71
12	68	5.05 ± 0.92	68	56.53 ± 4.10	71	4.78 ± 1.02	69	57.47 ± 4.54
13	82	5.29 ± 1.19	80	58.99 ± 4.58	55	5.18 ± 1.24	54	58.12 ± 5.55
14	60	5.44 ± 1.20	58	58.94 ± 5.49	74	5.07 ± 1.07	75	56.39 ± 4.90
15	62	5.63 ± 1.11	61	60.1 ± 4.74	52	5.25 ± 1.05	52	59.6 ± 3.86
16	81	6.04 ± 1.19	77	61.25 ± 4.93	71	5.51 ± 1.15	70	58.31 ± 4.69
17	63	5.78 ± 1.12	63	59.61 ± 4.98	70	5.65 ± 1.29	69	60.25 ± 4.64
18	59	5.94 ± 1.04	58	61.04 ± 4.50	59	5.57 ± 1.01	58	60.01 ± 3.53
19	62	6.2 ± 1.22	65	62.08 ± 4.08	70	5.77 ± 1.22	68	59.93 ± 5.1
20	65	6.07 ± 1.14	66	60.98 ± 4.53	53	5.77 ± 1.24	53	60.46 ± 4.14
21	58	6.52 ± 0.89	59	63.92 ± 4.21	67	6.04 ± 1.13	66	62.52 ± 4.02
22	59	6.29 ± 1.01	60	62.68 ± 4.32	68	6.18 ± 1.15	68	62.40 ± 4.32
23	58	6.47 ± 1.30	58	63.08 ± 5.73	36	6.29 ± 1.14	35	63.10 ± 3.32
24	47	6.47 ± 1.32	47	63.56 ± 4.67	43	6.32 ± 1.49	42	63.55 ± 6.67
25	54	6.68 ± 1.20	53	64.91 ± 4.05	41	6.06 ± 1.08	42	60.35 ± 4.81
26	30	6.92 ± 1.41	30	65.07 ± 4.00	56	6.19 ± 1.25	56	63.54 ± 6.07
Overall	1768	5.08 ± 1.48	1759	57.33 ± 6.57	1772	4.8 ± 1.43	1756	56.43 ± 6.53

Detailed tables presenting percentiles for Pakistani standards, along with corresponding values of lambda $\left(\lambda \right), mu \left(\mu \right), sigma \left(\sigma \right),\, and\, tau \left(\tau \right)$ for each age and fitted model, can be found in the supplementary material (refer to Tables S9-S12).

The selected degrees of freedom for L, M, and S were (0, 1, 0) for both genders in W/A, and (0, 1, 0) for males in H/A. When developing H/A curves for females, the optimal degrees of freedom were (1, 1, 1). Polynomial smoothing was applicable to only one model with degrees of freedom (1, 1, 1) and not for the other three. The preferred family for all four curves was BCPE, utilizing a penalized spline (ps) as the smoothing method, as indicated by the lowest BIC value detailed in Table 2.

**Table 2:** Selection of Optimum Degrees of Freedom and Optimum Model (Family and Smoothing Method) for each of the Four Curves using RefCurve_0.4.2 Software

		Weight for age		Height for age	
		Male	Female	Male	Female
		Optimal Degrees of freedom for L, M & S			
Parameters (Test range)	L (0–3)	0	0	0	1
	M (0–3)	1	1	1	1
	S (0–3)	0	0	0	1
	BIC	5059.28	4972.354	10420.34	10555.5
**Family**	**Smoothing**	**Bayesian Information Criteria (BIC)**			
BCCG	ps	5059.928	4972.354	10420.34	10555.5
	cs	5059.999	4972.414	10420.43	10555.63
	poly	NA	NA	NA	10584.31
**BCPE**	**ps**	**5053.671**	**4936.895**	**10296.73**	**10404.86**
	cs	5053.767	4936.966	10296.86	10405.01
	poly	NA	NA	NA	10441.63
BCT	ps	5058.059	4945.884	10314.07	10431.31
	cs	5058.145	4945.958	10314.22	10431.49
	poly	NA	NA	NA	10461.1
**Value of** $\varvec{\tau }$ ***for selected models***	**BCPE-ps**	**1.660418**	**1.448022**	**1.188859**	**1.114607**

Note: NA stands for not applicable as the model does not converge if polynomial is used with df = 0 for any of the parameter of location, shape and scale

**Fig. 2:**
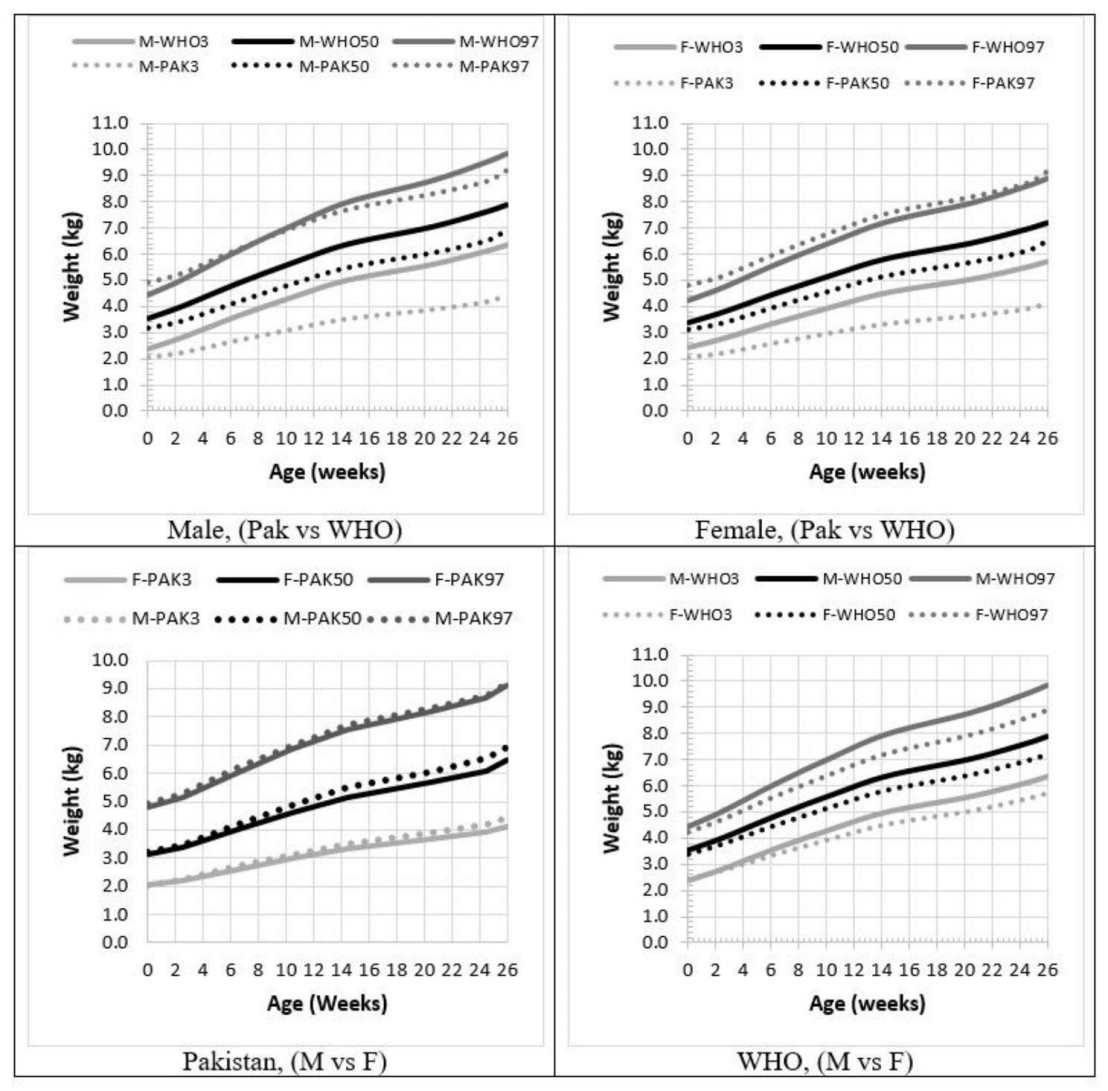
Indigenous growth standards of Pakistani children in comparison to WHO standards (W/A)

The growth charts for Weight-for-Age (W/A) based on the best-fitted models are illustrated in Fig. 2. Notably, the 97th percentile for the indigenous Pakistani distribution closely aligns with the WHO standard. However, a distinction arises with the median values. While WHO’s median starts at approximately 3.5 kg, Pakistan’s median commences at 3.15 kg. As age progresses, this discrepancy widens. By 26 weeks, there is an almost 1-kilogram gap between the medians of the WHO and Pakistani standards, with the Pakistani standard being lower. The most significant disparity is observed in the 3rd percentile between the two standards. Similar to the median, the difference is narrower in the early days after birth but widens notably with age. At 6 months, the difference is nearly 2 kg. The 3rd percentile for Pakistani standards concludes at 4.5 kg, in contrast to the WHO standard, which reaches almost 6.3 kg. In terms of female standards, the disparities with WHO females are smaller compared to males. In fact, Pakistani females exhibit higher upper percentiles from the outset to the conclusion, albeit the gap diminishes with advancing age. The trajectories of median and 3rd percentile for Pakistani standards consistently remain below those of the WHO standards. The difference in medians at 6 months is approximately 0.7 kg, while for the 3rd percentile, it is observed to be 1.6 kg. Furthermore, a comparative analysis between males and females from both standards reveals that Pakistani males exhibit growth patterns quite similar to Pakistani females. However, WHO standards exhibit a clear distinction in favor of males. (see Fig. 2).

In the case of Height-for-Age (H/A) comparisons, a distinct pattern emerges. The Pakistani standards exhibit broader ranges on both extremes in comparison to the WHO standards. For boys, the upper percentiles are nearly identical or display minimal differences. On the other hand, for girls, the Pakistani standards reflect an additional 6 centimeters in height at the 97th percentile in comparison to WHO standards at birth, and 4.6 centimeters at 6 months. While the median height remains lower for Pakistani standards, the difference does not exceed 2 centimeters at any given point between birth and 6 months. However, the 3rd percentile is notably lower for Pakistani standards, encompassing both genders consistently. At 6 months of age, the disparity for females is 9.2 centimeters, while for males, it is 9.6 centimeters.

**Fig. 3:**
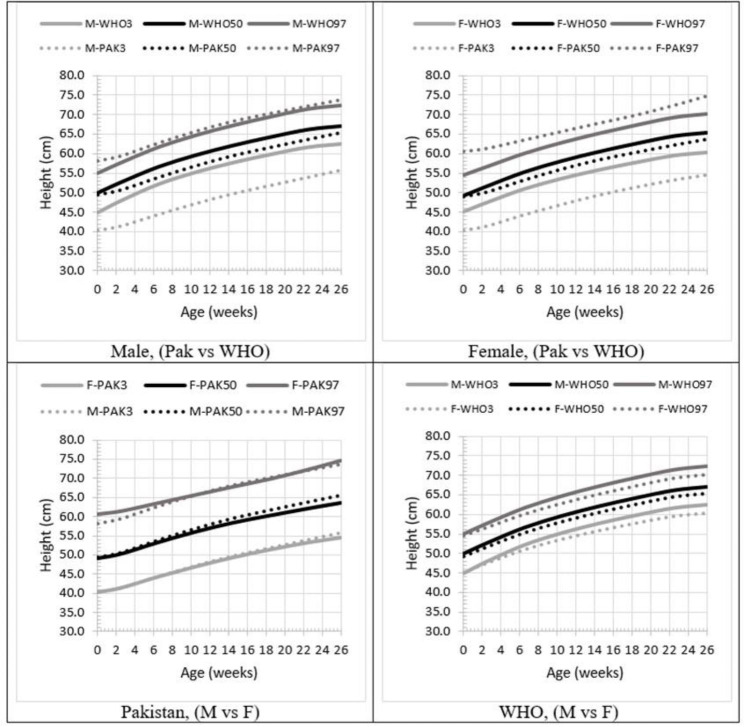
Indigenous growth standards of Pakistani children in comparison to WHO standards (H/A)

A similar trend emerged when comparing the two genders in the Pakistani Height-for-Age (H/A) chart. Only a slight variation was noted at birth, where females displayed 1.2 centimeters more height for the 97th percentile. For the median, boys exhibited approximately 1.8 centimeters more height than girls, but the curves closely aligned for both genders at the 3rd percentile. Conversely, the WHO standards demonstrated a clear widening gap between males and females as age increased. At six months, the difference for the 97th percentile was 2.3 centimeters, 1.7 centimeters for the median, and 2.2 centimeters for the 3rd percentile. On average, males tend to be taller than females at six months of age. (Fig. 3)

Another study recently published for Pakistan is based on the data from national nutritional survey data. The results of 3rd, 50th and 97th percentiles of this study are also compared with that study. (Figure S1, supplementary material)

## Discussion

Pakistan has yet to develop specific growth charts tailored to exclusively breastfed children aged 0–6 months [3–6]. Creating indigenous growth charts for a country is a complex and challenging endeavor. The initial step involved careful case selection, building upon prior research [13]. In this study, exclusive breastfeeding was a key nutritional criterion, as detailed in the initial section of the methodology and further expounded upon in [13]. An additional refinement was introduced by setting the condition that no more than two adults should share a sleeping room to filter out congested households. This selection criteria aligns closely with the criteria applied in an Indian study [14] and WHO standards [16, 17].

The WHO employed BCPE with cubic spline or polynomial smoothing techniques for charts covering 0–5 years [17]. In our study, following AIC criteria, BCPE emerged as the superior method, but with penalized spline (ps) as the chosen smoothing technique. Notably, the AIC for BCPE with cubic spline showed only marginal differences, extending to the first and second decimal places. For boys’ W/A, girls’ W/A, and boys’ H/A, the degrees of freedom were (L = 0, M = 1, S = 0), whereas for girls’ H/A, they were (L = 1, M = 1, S = 1). In the WHO study [17], these degrees of freedom were (L = 2, M = 11, S = 7), (L = 3, M = 11, S = 7), (L = 0, M = 12, S = 6), and (L = 0, M = 10, S = 5) respectively, with τ fixed at 2 for all four charts [24]. The Indian study employed degrees of freedom as (L = 1, M = 10, S = 9), (L = 2, M = 10, S = 9), (L = 0, M = 11, S = 9), and (L = 0, M = 9, S = 8) respectively, with a consistent τ value of 2 [14]. In our study, the calculated τ values for the four models were 1.660, 1.448, 1.189, and 1.115 respectively. The variance in degrees of freedom may be attributed in part to differences in age range, as the WHO and Indian studies focused on children aged 0–5 years, while our study centers on exclusively breastfed children aged 0–6 months [14, 24].

After fitting the models and generating the growth charts, our first step was to compare them with the WHO charts. To facilitate this, we downloaded the WHO percentiles for 0–3 years in Excel format and extracted the values corresponding to the specified percentiles. The provided WHO charts furnished values for ages 0, 15, 45, 75, 105, 145, 175, and 205 days. To establish comparability, we applied interpolation to derive measurements for all four parameters at 175 and 205 days, thus obtaining values for the age of 180 days (6 months). Subsequently, we created graphs for comparison.

At birth, the Pakistani standards indicated a median weight of 3.19 kg for boys, while the WHO median was 3.53 kg. The 3rd and 97th percentiles for the two charts were (2.05 vs. 2.36) and (4.91 vs. 4.45) respectively. This suggests that the Pakistani standards demonstrated a higher 97th percentile compared to the WHO at birth, but lower values in terms of median and 3rd percentile. These discrepancies persisted and even increased with time, such that at 6 months of age, the Pakistani standards exhibited lower values than the WHO standards, with measurements of (6.95 vs. 7.90) for median and (4.45 vs. 6.33) for 3rd percentile. The 97th percentile for Pakistan and WHO overlapped between the ages of 6 and 10 weeks, after which it began to diverge. By 6 months of age, the 97th percentiles for Pakistan and WHO were (9.2 vs. 9.84) respectively.

In India, according to Khadilker et al. 2019 [25], synthetic growth charts were developed, and their study reported a median weight of 2.9 kg for both boys and girls at birth. At 6 months, these measures were 6.4 for boys and 6.5 for girls. Thus, the medians at birth for Pakistan, India, and WHO were (3.19, 2.90, 3.53), and at 6 months were (6.95, 6.40, 7.90) for boys. For girls, the medians at birth and 6 months were (3.12, 2.90, 3.40) and (6.48, 6.5, 7.21) respectively.

In terms of H/A, the baseline median for boys stood at (49.5 for Pakistan, 50.8 for India, and 50.0 for WHO), and for girls, it was (49.1 for Pakistan, 50.7 for India, and 49.3 for WHO). Since the synthetic growth charts [25] were designed for children aged 0 to 18 years, data points for the 0–6 month range were unavailable, making it impossible to plot these values.

It is evident that W/A showed a lower average compared to WHO but a better performance than India. While the patterns in Pakistani, Indian, and WHO charts align, they exhibit distinct trajectories. The Pakistani charts demonstrated a broader range than WHO, with both the 3rd and 97th percentiles being lower and higher respectively, for both boys and girls. At birth, the 97th percentile for girls in (Pakistan, India, and WHO) was (60.6, 54.6, and 54.5) while the 3rd percentile was (40.3, 46.7, and 47.5).

The most recent study published in 2023 provides reference charts based on the national nutritional survey for children aged 0–60 months in Pakistan [26]. A comparison between our study and this recent work reveals that the median H/A for males at 1 month is 52.35 compared to 52.77, at 3 months it’s 58.94 compared to 58.88, and at 6 months it’s 65.44 compared to 65.26. For females, the comparison shows that the H/A at 1, 3, and 6 months are (51.80 vs. 52.09), (57.87 vs. 57.99), and (63.62 vs. 63.78) respectively.

Similarly, when comparing W/A for males, the respective pairs of values at 1, 3, and 6 months were (3.84 vs. 4.07), (5.36 vs. 5.36), and (6.95 vs. 6.93). For females, the values were (3.69 vs. 4.02), (5.04 vs. 5.09), and (6.48 vs. 6.45) [26].

In this comparison, the median values and 3rd percentiles almost coincide. However, the 97th percentiles of the other study are slightly higher than in our study when comparing W/A. When comparing H/A, it’s observed that the median and 97th percentile coincide for both studies, but the 3rd percentile in our study is slightly lower [26].

These differences may arise due to several factors: (1) our growth curves are developed for data of children up to 6 months (26 weeks) old, whereas the other study is based on data up to 5 years old; (2) the two datasets used are different; [26] (3) Our study provides estimates for 1-week intervals, while the other is for 3-month intervals. Our study may have an advantage as growth during the first six months of age is at a rapid rate and merits detailed study at smaller age intervals.

Another important observation in this study was the minimal difference observed between males and females in Pakistani standards for children under the age of 6 months, whether considering W/A or H/A. In contrast, the WHO charts exhibited a distinct difference in trajectory when analyzed for W/A, while H/A also displayed a difference, though it was not as pronounced between the two genders.

When comparing our study with the systematic review presented by Natale [11], we find that Pakistani standards align with the lowest trajectory for those four metrics, namely Saudi Arabia, Japan breastfed, India, and Japan All, in comparison to MGRS for W/A in boys. Although we have not specifically calculated the average weight at 2 years, the trends indicate a similarity. This suggests a need for further research on Pakistani standards, particularly up to the age of two years and beyond.

This study specifically focuses on Height-for-Age (H/A) and Weight-for-Age (W/A) as key anthropometric measures. It’s important to note that data pertaining to other anthropometric measures is not included in the MICS dataset. But due to large disparity in the reference values of other anthropometric characteristics i.e., BMI, neck circumference, head and mid-upper arm circumference etc. [27–30] for Pakistani children and adolescents with the international studies, it is highly recommended that reference data of these indicators should be published for a more comprehensive evaluation of nutritional status among Pakistani infants.

## Conclusion

The findings of this study underscore the significance of the Novel Case Selection Method in crafting growth standards tailored to the unique needs of lower and middle-income countries. Leveraging the wealth of data from the extensive MICS survey, which provides a comprehensive snapshot of the nation’s demographics, this study adeptly addressed the task of assembling a representative national sample. The methodology also took into account specific contextual nuances crucial for establishing accurate standards. Consequently, the growth charts generated through this approach offer substantial practical value and warrant validation through established cross-sectional and longitudinal data sources. This achievement stands as an encouraging precedent for policy makers and clinicians seeking to develop their own homegrown growth charts, providing a vital tool for monitoring child health and nutrition in diverse global contexts.

### Electronic supplementary material

Below is the link to the electronic supplementary material.

Supplementary Material 1

## Data Availability

Data used and analyzed in the preparation of this article were obtained from the UNICEF website, https://mics.unicef.org/surveys. Data used is of MICS round 6 for different provinces of Pakistan.
