# Supplementary material for: Height-for-age and weight-for-age growth charts for Pakistani infants under six months: derived from a novel case selection method using multiple indicator cluster survey data
Source: BMC Med Res Methodol. 2023 Dec 8;23:289. doi: 10.1186/s12874-023-02116-y (PMC10709855; doi:10.1186/s12874-023-02116-y)
Supplement: Supplementary file 1 — Supplementary Material 1 [file 12874_2023_2116_MOESM1_ESM.docx]

Table S1: Attributes of liquid items ingested by children within the past 24 hours, featuring variable names in the dataset and designated names for algorithms.

| MICS file  name | designated  name | Characteristic explained |
| --- | --- | --- |
| BD4 | $x_{1}$ | Child drank anything from a bottle with a nipple yesterday |
| BD5 | $x_{2}$ | Child drank ORS yesterday |
| BD6 | $x_{3}$ | Child drank or ate vitamin or mineral supplements yesterday |
| BD7A | $x_{4}$ | Child drank plain water yesterday |
| BD7B | $x_{5}$ | Child drank juice or juice drinks yesterday |
| BD7C | $x_{6}$ | Child drank clear broth or clear soup yesterday |
| BD7D | $x_{7}$ | Child drank infant formula yesterday |
| BD7E | $x_{8}$ | Child drank milk yesterday |
| BD7X | $x_{9}$ | Child drank any other liquid yesterday |

Table S2: Attributes of solids and semi solids consumed by children within the past 24 hours, featuring variable names in the dataset and designated names for algorithms.

| MCS File names | Designated  names | Characteristics of item consumed |
| --- | --- | --- |
| BD8A | $y_{1}$ | Child drank or ate yogurt yesterday |
| BD8B | $y_{2}$ | Child ate fortified baby food cerelac etc. yesterday |
| BD8C | $y_{3}$ | Child ate foods made from grains yesterday |
| BD8D | $y_{4}$ | Child ate pumpkin, carrots, squash etc. yesterday |
| BD8E | $y_{5}$ | Child ate white potatoes, white yams, manioc, cassava etc. yesterday |
| BD8F | $y_{6}$ | Child ate green leafy vegetables yesterday |
| BD8G | $y_{7}$ | Child ate ripe mangoes, papayas, apricots etc. yesterday |
| BD8H | $y_{8}$ | Child ate other fruits or vegetables yesterday |
| BD8I | $y_{9}$ | Child ate liver, kidney, heart or other organ meat yesterday |
| BD8J | $y_{10}$ | Child ate meat, as beef, lamb, goat, chicken, duck etc. or sausages made from these meats yesterday |
| BD8K | $y_{11}$ | Child ate eggs yesterday |
| BD8L | $y_{12}$ | Child ate fresh or dried fish or shellfish yesterday |
| BD8M | $y_{13}$ | Child ate beans, peas, lentils or nuts or any food made from these yesterday |
| BD8N | $y_{14}$ | Child ate cheese or other food made from milk yesterday |
| BD8X | $y_{15}$ | Child ate other solid, semi-solid or soft food yesterday |

**Cross validation was done by 70:30 split and training and testing, both data sets, had same optimal model selection mostly and if differentiated the difference was almost negligible.**

**The selected model on after evaluating 4 models on all three criteria (Global Deviance, AIC and SBC), was the BCPE with penalized splines (ps)**

Table S3 Cross validation for Height-for-Age female through 70:30 split for selected optimum degrees of freedom

| Family/Criteria | | Training | | | Testing | | |
| --- | --- | --- | --- | --- | --- | --- | --- |
|  |  | df(M) = 1, df (S) = 1, df(L) = 1 | | | df(M) = 1, df (S) = 1, df(L) = 1 | | |
| BCCG |  | ps | cs | poly | ps | cs | poly |
|  | GD | 7245.871 | 7245.943 | 7284.209 | 3225.793 | 3225.833 | 3247.85 |
|  | AIC | 7263.871 | 7263.941 | 7296.209 | 3243.793 | 3243.832 | 3259.85 |
|  | SBC | 7309.823 | 7309.888 | 7326.844 | 3282.367 | 3282.402 | 3285.566 |
| BCPE | GD | **7141.614** | 7141.715 | 7182.688 | **3178.161** | 3178.240 | 3202.586 |
|  | AIC | **7161.614** | 7161.713 | 7196.688 | **3198.161** | 3198.238 | 3216.586 |
|  | SBC | **7212.672** | 7212.766 | 7232.428 | **3241.021** | 3241.094 | 3246.588 |
| BCT | GD | 7155.875 | 7156.001 | 7192.008 | 3190.674 | 3190.713 | 3213.299 |
|  | AIC | 7175.875 | 7175.999 | 7206.008 | 3210.674 | 3210.711 | 3227.299 |
|  | SBC | 7226.933 | 7227.052 | 7241.748 | 3253.534 | 3253.568 | 3257.301 |

The training data and testing data both resulted “BCPE” with penalized spline (ps) as smoothing tool with a very thin margin to cubic splines “cs” as smoothing tool.

**Final presentation on AIC was “BCPE” with “ps”**

Table S4 Cross validation for H/A male through 70:30 split for selected optimum degrees of freedom

| Family/Criteria | | Training | | | Testing | | |
| --- | --- | --- | --- | --- | --- | --- | --- |
|  |  | df(M) = 1, df (S) = 0, df(L) = 0 | | | df(M) = 1, df (S) = 0, df(L) = 0 | | |
| BCCG |  | ps | cs | poly | ps | cs | poly |
|  | GD | 7048.051 | 7048.095 | NA | 3316.014 | 3316.048 | NA |
|  | AIC | 7062.051 | 7062.094 | NA | 3330.014 | 3330.047 | NA |
|  | SBC | 7097.67 | 7097.711 | NA | 3360.322 | 3360.354 | NA |
| BCPE | GD | **6970.426** | 6970.498 | NA | **3259.012** | 3259.125 | NA |
|  | AIC | **6986.426** | 6986.497 | NA | **3275.012** | 3275.124 | NA |
|  | SBC | **7027.133** | 7027.202 | NA | **3309.65** | 3309.76 | NA |
| BCT | GD | 6977.602 | 6977.676 | NA | 3271.308 | 3271.371 | NA |
|  | AIC | 6993.602 | 6993.675 | NA | 3287.308 | 3287.371 | NA |
|  | SBC | 7034.309 | 7034.38 | NA | 3321.946 | 3322.007 | NA |

The training data and testing data both resulted “BCPE” with penalized spline (ps) as smoothing tool with a very thin margin to cubic splines “cs” as smoothing tool.

**Final presentation on AIC was “BCPE” with “ps”**

Table S5 Cross validation for W/A female through 70:30 split for selected optimum degrees of freedom

| Family/Criteria | | Training | | | Testing | | |
| --- | --- | --- | --- | --- | --- | --- | --- |
|  |  | df(M) = 1, df (S) = 0, df(L) = 0 | | | df(M) = 1, df (S) = 0, df(L) = 0 | | |
| BCCG |  | ps | cs | poly | ps | cs | poly |
|  | GD | 3542.354 | 3542.418 | NA | 1366.982 | 1366.968 | NA |
|  | AIC | 3556.354 | 3556.418 | NA | 1380.982 | 1380.969 | NA |
|  | SBC | 3592.410 | 3592.471 | NA | 1410.442 | 1410.430 | NA |
| BCPE | GD | **3510.100** | 3510.169 | NA | 1357.809 | **1357.794** | NA |
|  | AIC | **3526.100** | 3526.168 | NA | 1373.809 | **1373.795** | NA |
|  | SBC | **3567.306** | 3567.372 | NA | 1407.478 | **1407.465** | NA |
| BCT | GD | 3512.760 | 3512.834 | NA | 1362.741 | 1362.731 | NA |
|  | AIC | 3528.760 | 3528.833 | NA | 1378.741 | 1378.731 | NA |
|  | SBC | 3569.966 | 3570.037 | NA | 1412.410 | 1412.401 | NA |

The training data resulted in selection of “BCPE” with penalized spline (ps) as smoothing tool with a very thin margin to cubic splines “cs” as smoothing tool. While the testing data resulted in selection of “BCPE” with cubic spline as smoothing tool as compared to penalized spline with very thin margin.

**Final presentation on AIC was “BCPE” with “ps”**

Table S6 Cross validation for W-A male through 70:30 split for selected optimum degrees of freedom

| Family/Criteria | | Training | | | Testing | | |
| --- | --- | --- | --- | --- | --- | --- | --- |
|  |  | df(M) = 1, df (S) = 0, df(L) = 0 | | | df(M) = 1, df (S) = 0, df(L) = 0 | | |
| BCCG |  | ps | cs | poly | ps | cs | poly |
|  | GD | 3561.832 | 3561.908 | NA | 1430.09 | 1430.088 | NA |
|  | AIC | 3575.832 | 3575.907 | NA | **1444.09** | 1444.089 | NA |
|  | SBC | 3611.870 | 3611.944 | NA | **1473.536** | 1473.536 | NA |
| BCPE | GD | **3551.352** | 3551.463 | NA | **1428.605** | 1428.606 | NA |
|  | AIC | **3567.352** | 3567.463 | NA | **1444.605** | 1444.607 | NA |
|  | SBC | **3608.539** | 3608.647 | NA | 1478.257 | 1478.261 | NA |
| BCT | GD | 3554.762 | 3554.862 | NA | 1429.763 | 1429.762 | NA |
|  | AIC | 3570.762 | 3570.861 | NA | 1445.763 | 1445.763 | NA |
|  | SBC | 3611.949 | 3612.046 | NA | 1479.416 | 1479.417 | NA |

Here the training data resulted in selection of “BCPE” with penalized spline (ps) as smoothing tool with a very thin margin to cubic splines “cs” as smoothing tool. While the testing data resulted in selection of “BCPE” with “ps” for global deviance and “BCCG” with “ps” as smoothing technique with no difference from “cs” either on AIC or SBC. The “BCPE” and “BCCG” also had a very thin margin for AIC.

**Final presentation on AIC was “BCPE” with “ps”**

Table S7 Selection of optimum degrees of freedom on BIC and optimum model for each of the four curves on three criteria (Global deviance, AIC and SBC)

|  | | DF for | Male (Weight) | Female (Weight) | Male (Height) | Female (Height) |
| --- | --- | --- | --- | --- | --- | --- |
|  |  | L | 0 | 0 | 0 | 1 |
|  |  | M | 1 | 1 | 1 | 1 |
|  |  | S | 0 | 0 | 0 | 1 |
| Family | smoothing | Criteria | Values for each criteria | | | |
| BCCG | ps | GD | 5007.585 | 4919.995 | 10368.03 | 10488.26 |
|  |  | AIC | 5021.585 | 4933.995 | 10382.03 | 10506.26 |
|  |  | SBC | 5059.928 | 4972.354 | 10420.34 | 10555.5 |
|  | cs | GD | 5007.659 | 4920.057 | 10368.12 | 10488.4 |
|  |  | AIC | 5021.658 | 4934.057 | 10382.12 | 10506.4 |
|  |  | SBC | 5059.999 | 4972.414 | 10420.43 | 10555.63 |
|  | poly | GD | NA | NA | NA | 10539.48 |
|  |  | AIC | NA | NA | NA | 10551.48 |
|  |  | SBC | NA | NA | NA | 10584.31 |
| BCPE | **ps** | **GD** | **4993.85** | **4877.055** | **10236.95** | **10330.15** |
|  |  | **AIC** | **5009.85** | **4893.055** | **10252.95** | **10350.15** |
|  |  | **SBC** | **5053.671** | **4936.895** | **10296.73** | **10404.86** |
|  | cs | GD | 4993.95 | 4877.13 | 10237.08 | 10330.31 |
|  |  | AIC | 5009.949 | 4893.129 | 10253.08 | 10350.3 |
|  |  | SBC | 5053.767 | 4936.966 | 10296.86 | 10405.01 |
|  | poly | GD | NA | NA | NA | 10389.33 |
|  |  | AIC | NA | NA | NA | 10403.33 |
|  |  | SBC | NA | NA | NA | 10441.63 |
| BCT | ps | GD | 4998.237 | 4886.045 | 10254.29 | 10356.61 |
|  |  | AIC | 5014.237 | 4902.045 | 10270.29 | 10376.61 |
|  |  | SBC | 5058.059 | 4945.884 | 10314.07 | 10431.31 |
|  | cs | GD | 4998.328 | 4886.121 | 10254.44 | 10356.79 |
|  |  | AIC | 5014.327 | 4902.121 | 10270.44 | 10376.78 |
|  |  | SBC | 5058.145 | 4945.958 | 10314.22 | 10431.49 |
|  | poly | GD | NA | NA | NA | 10408.81 |
|  |  | AIC | NA | NA | NA | 10422.81 |
|  |  | SBC | NA | NA | NA | 10461.1 |

**NA stands for not applicable**

Table S8 Distribution of selected cases by province, rural urban residence and gender

| **Province** | **Residential status** | **Sex** | | | | | |
| --- | --- | --- | --- | --- | --- | --- | --- |
|  |  | **MALE** | | **FEMALE** | | **Total** | |
|  |  | **Count** | **%** | **Count** | **%** | **Count** | **%** |
| **KPK** | **URBAN** | 46 | 1.3 | 50 | 1.4 | 96 | 2.6 |
|  | **RURAL** | 359 | 9.8 | 386 | 10.6 | 745 | 20.4 |
| **Punjab** | **URBAN** | 184 | 5.0 | 175 | 4.8 | 359 | 9.8 |
|  | **RURAL** | 577 | 15.8 | 528 | 14.4 | 1105 | 30.2 |
| **Sindh** | **URBAN** | 150 | 4.1 | 154 | 4.2 | 304 | 8.3 |
|  | **RURAL** | 239 | 6.5 | 276 | 7.6 | 515 | 14.1 |
| **Balochistan** | **URBAN** | 43 | 1.2 | 62 | 1.7 | 105 | 2.9 |
|  | **RURAL** | 233 | 6.4 | 193 | 5.3 | 426 | 11.7 |
| **Total** | **URBAN** | 423 | 11.6 | 441 | 12.1 | 864 | 23.6 |
|  | **RURAL** | 1408 | 38.5 | 1383 | 37.8 | 2791 | 76.4 |
|  | **Total** | **1831** | **50.1** | **1824** | **49.9** | **3655** | **100.0** |
| **Age (weeks)** | **Mean ± SD** | 12.27 ± 7.27 | | 12.00 ± 7.45 | | 12.14 ± 7.36 | |
| **Weight (kg)** | **Mean ± SD** | 5.18 ± 1.89 | | 4.83 ± 1.67 | | 5.00 ± 1.79 | |
| **Height (cm)** | **Mean ± SD** | 57.46 ± 7.78 | | 56.33 ± 7.75 | | 56.89 ± 7.79 | |

Table S9 Presentation of meu, sigma, lambda for each age and tau for H/A females curve with percentiles **(BCPE, ps, df (L=1, M=1, S=1))**

|  | $\boldsymbol{\mu}$ | $\boldsymbol{\sigma}$ | $\boldsymbol{\lambda}$ | $\boldsymbol{\tau}$ | x | C3 | C10 | C25 | C50 | C75 | C90 | C97 |
| --- | --- | --- | --- | --- | --- | --- | --- | --- | --- | --- | --- | --- |
| 1 | 49.09 | 0.10 | -0.35 | 1.11 | 1.00 | 40.32 | 43.63 | 46.54 | 49.09 | 51.83 | 55.51 | 60.63 |
| 2 | 49.28 | 0.10 | -0.32 | 1.11 | 1.25 | 40.51 | 43.83 | 46.74 | 49.28 | 52.02 | 55.68 | 60.76 |
| 3 | 49.48 | 0.10 | -0.30 | 1.11 | 1.51 | 40.69 | 44.02 | 46.93 | 49.48 | 52.21 | 55.85 | 60.89 |
| 4 | 49.67 | 0.10 | -0.27 | 1.11 | 1.76 | 40.88 | 44.22 | 47.13 | 49.67 | 52.39 | 56.02 | 61.02 |
| 5 | 49.87 | 0.10 | -0.25 | 1.11 | 2.01 | 41.07 | 44.41 | 47.33 | 49.87 | 52.58 | 56.19 | 61.15 |
| 6 | 50.06 | 0.10 | -0.22 | 1.11 | 2.26 | 41.25 | 44.61 | 47.53 | 50.06 | 52.77 | 56.36 | 61.28 |
| 7 | 50.26 | 0.10 | -0.19 | 1.11 | 2.52 | 41.44 | 44.80 | 47.72 | 50.26 | 52.96 | 56.53 | 61.42 |
| 8 | 50.45 | 0.10 | -0.17 | 1.11 | 2.77 | 41.62 | 45.00 | 47.92 | 50.45 | 53.14 | 56.70 | 61.55 |
| 9 | 50.65 | 0.10 | -0.14 | 1.11 | 3.02 | 41.81 | 45.19 | 48.12 | 50.65 | 53.33 | 56.87 | 61.68 |
| 10 | 50.84 | 0.10 | -0.12 | 1.11 | 3.27 | 41.99 | 45.39 | 48.31 | 50.84 | 53.51 | 57.04 | 61.82 |
| 11 | 51.03 | 0.10 | -0.09 | 1.11 | 3.53 | 42.18 | 45.58 | 48.51 | 51.03 | 53.70 | 57.21 | 61.95 |
| 12 | 51.23 | 0.10 | -0.06 | 1.11 | 3.78 | 42.36 | 45.77 | 48.71 | 51.23 | 53.89 | 57.38 | 62.09 |
| 13 | 51.42 | 0.10 | -0.04 | 1.11 | 4.03 | 42.55 | 45.97 | 48.90 | 51.42 | 54.07 | 57.54 | 62.22 |
| 14 | 51.61 | 0.10 | -0.01 | 1.11 | 4.28 | 42.73 | 46.16 | 49.10 | 51.61 | 54.26 | 57.71 | 62.36 |
| 15 | 51.80 | 0.09 | 0.02 | 1.11 | 4.54 | 42.92 | 46.35 | 49.29 | 51.80 | 54.44 | 57.88 | 62.49 |
| 16 | 51.99 | 0.09 | 0.04 | 1.11 | 4.79 | 43.10 | 46.54 | 49.48 | 51.99 | 54.62 | 58.05 | 62.62 |
| 17 | 52.18 | 0.09 | 0.07 | 1.11 | 5.04 | 43.28 | 46.73 | 49.68 | 52.18 | 54.81 | 58.21 | 62.76 |
| 18 | 52.37 | 0.09 | 0.10 | 1.11 | 5.29 | 43.46 | 46.92 | 49.87 | 52.37 | 54.99 | 58.38 | 62.89 |
| 19 | 52.56 | 0.09 | 0.13 | 1.11 | 5.55 | 43.64 | 47.11 | 50.06 | 52.56 | 55.17 | 58.55 | 63.03 |
| 20 | 52.75 | 0.09 | 0.16 | 1.11 | 5.80 | 43.82 | 47.30 | 50.25 | 52.75 | 55.35 | 58.71 | 63.16 |
| 21 | 52.93 | 0.09 | 0.19 | 1.11 | 6.05 | 43.99 | 47.49 | 50.44 | 52.93 | 55.53 | 58.87 | 63.30 |
| 22 | 53.12 | 0.09 | 0.21 | 1.11 | 6.30 | 44.17 | 47.67 | 50.62 | 53.12 | 55.71 | 59.04 | 63.43 |
| 23 | 53.30 | 0.09 | 0.24 | 1.11 | 6.56 | 44.34 | 47.86 | 50.81 | 53.30 | 55.88 | 59.20 | 63.56 |
| 24 | 53.48 | 0.09 | 0.27 | 1.11 | 6.81 | 44.52 | 48.04 | 50.99 | 53.48 | 56.06 | 59.36 | 63.70 |
| 25 | 53.66 | 0.09 | 0.30 | 1.11 | 7.06 | 44.69 | 48.22 | 51.18 | 53.66 | 56.23 | 59.52 | 63.83 |
| 26 | 53.84 | 0.09 | 0.33 | 1.11 | 7.31 | 44.86 | 48.40 | 51.36 | 53.84 | 56.41 | 59.68 | 63.97 |
| 27 | 54.02 | 0.09 | 0.36 | 1.11 | 7.57 | 45.03 | 48.58 | 51.54 | 54.02 | 56.58 | 59.84 | 64.10 |
| 28 | 54.20 | 0.09 | 0.38 | 1.11 | 7.82 | 45.20 | 48.76 | 51.72 | 54.20 | 56.75 | 60.00 | 64.23 |
| 29 | 54.38 | 0.09 | 0.41 | 1.11 | 8.07 | 45.36 | 48.93 | 51.90 | 54.38 | 56.92 | 60.16 | 64.37 |
| 30 | 54.55 | 0.09 | 0.44 | 1.11 | 8.32 | 45.53 | 49.11 | 52.08 | 54.55 | 57.09 | 60.32 | 64.50 |
| 31 | 54.73 | 0.09 | 0.47 | 1.11 | 8.58 | 45.69 | 49.28 | 52.25 | 54.73 | 57.26 | 60.48 | 64.64 |
| 32 | 54.90 | 0.09 | 0.49 | 1.11 | 8.83 | 45.85 | 49.45 | 52.43 | 54.90 | 57.43 | 60.63 | 64.77 |
| 33 | 55.07 | 0.09 | 0.52 | 1.11 | 9.08 | 46.01 | 49.62 | 52.60 | 55.07 | 57.59 | 60.79 | 64.91 |
| 34 | 55.24 | 0.09 | 0.54 | 1.11 | 9.33 | 46.17 | 49.79 | 52.77 | 55.24 | 57.76 | 60.94 | 65.04 |
| 35 | 55.40 | 0.09 | 0.56 | 1.11 | 9.59 | 46.33 | 49.96 | 52.94 | 55.40 | 57.92 | 61.09 | 65.17 |
| 36 | 55.57 | 0.09 | 0.59 | 1.11 | 9.84 | 46.49 | 50.12 | 53.10 | 55.57 | 58.08 | 61.25 | 65.31 |
| 37 | 55.73 | 0.09 | 0.61 | 1.11 | 10.09 | 46.65 | 50.29 | 53.27 | 55.73 | 58.24 | 61.40 | 65.44 |
| 38 | 55.90 | 0.08 | 0.63 | 1.11 | 10.34 | 46.80 | 50.45 | 53.43 | 55.90 | 58.40 | 61.55 | 65.58 |
| 39 | 56.06 | 0.08 | 0.65 | 1.11 | 10.60 | 46.96 | 50.61 | 53.60 | 56.06 | 58.56 | 61.70 | 65.71 |
| 40 | 56.22 | 0.08 | 0.66 | 1.11 | 10.85 | 47.11 | 50.77 | 53.76 | 56.22 | 58.71 | 61.85 | 65.85 |
| 41 | 56.38 | 0.08 | 0.68 | 1.11 | 11.10 | 47.27 | 50.93 | 53.92 | 56.38 | 58.87 | 61.99 | 65.98 |
| 42 | 56.53 | 0.08 | 0.70 | 1.11 | 11.35 | 47.42 | 51.09 | 54.08 | 56.53 | 59.02 | 62.14 | 66.11 |
| 43 | 56.69 | 0.08 | 0.71 | 1.11 | 11.61 | 47.57 | 51.24 | 54.23 | 56.69 | 59.17 | 62.29 | 66.25 |
| 44 | 56.84 | 0.08 | 0.72 | 1.11 | 11.86 | 47.72 | 51.40 | 54.39 | 56.84 | 59.32 | 62.43 | 66.38 |
| 45 | 56.99 | 0.08 | 0.74 | 1.11 | 12.11 | 47.87 | 51.55 | 54.54 | 56.99 | 59.47 | 62.57 | 66.51 |
| 46 | 57.14 | 0.08 | 0.75 | 1.11 | 12.36 | 48.02 | 51.70 | 54.69 | 57.14 | 59.62 | 62.71 | 66.65 |
| 47 | 57.29 | 0.08 | 0.75 | 1.11 | 12.62 | 48.17 | 51.85 | 54.84 | 57.29 | 59.76 | 62.86 | 66.78 |
| 48 | 57.44 | 0.08 | 0.76 | 1.11 | 12.87 | 48.32 | 52.00 | 54.99 | 57.44 | 59.91 | 63.00 | 66.91 |
| 49 | 57.58 | 0.08 | 0.77 | 1.11 | 13.12 | 48.47 | 52.15 | 55.14 | 57.58 | 60.05 | 63.13 | 67.04 |
| 50 | 57.73 | 0.08 | 0.77 | 1.11 | 13.37 | 48.61 | 52.30 | 55.28 | 57.73 | 60.19 | 63.27 | 67.18 |
| 51 | 57.87 | 0.08 | 0.77 | 1.11 | 13.63 | 48.76 | 52.44 | 55.43 | 57.87 | 60.33 | 63.41 | 67.31 |
| 52 | 58.01 | 0.08 | 0.78 | 1.11 | 13.88 | 48.91 | 52.59 | 55.57 | 58.01 | 60.47 | 63.55 | 67.44 |
| 53 | 58.15 | 0.08 | 0.78 | 1.11 | 14.13 | 49.05 | 52.73 | 55.71 | 58.15 | 60.61 | 63.68 | 67.57 |
| 54 | 58.29 | 0.08 | 0.77 | 1.11 | 14.38 | 49.20 | 52.87 | 55.85 | 58.29 | 60.75 | 63.82 | 67.71 |
| 55 | 58.42 | 0.08 | 0.77 | 1.11 | 14.64 | 49.34 | 53.01 | 55.99 | 58.42 | 60.88 | 63.95 | 67.84 |
| 56 | 58.56 | 0.08 | 0.76 | 1.11 | 14.89 | 49.49 | 53.15 | 56.12 | 58.56 | 61.02 | 64.08 | 67.97 |
| 57 | 58.69 | 0.08 | 0.76 | 1.11 | 15.14 | 49.63 | 53.29 | 56.26 | 58.69 | 61.15 | 64.22 | 68.11 |
| 58 | 58.82 | 0.08 | 0.75 | 1.11 | 15.39 | 49.77 | 53.43 | 56.39 | 58.82 | 61.28 | 64.35 | 68.24 |
| 59 | 58.96 | 0.08 | 0.74 | 1.11 | 15.65 | 49.91 | 53.56 | 56.53 | 58.96 | 61.41 | 64.48 | 68.38 |
| 60 | 59.09 | 0.08 | 0.73 | 1.11 | 15.90 | 50.05 | 53.70 | 56.66 | 59.09 | 61.54 | 64.61 | 68.51 |
| 61 | 59.22 | 0.08 | 0.72 | 1.11 | 16.15 | 50.19 | 53.83 | 56.79 | 59.22 | 61.67 | 64.74 | 68.65 |
| 62 | 59.34 | 0.08 | 0.70 | 1.11 | 16.40 | 50.33 | 53.96 | 56.92 | 59.34 | 61.80 | 64.87 | 68.79 |
| 63 | 59.47 | 0.08 | 0.69 | 1.11 | 16.66 | 50.46 | 54.09 | 57.04 | 59.47 | 61.93 | 65.01 | 68.92 |
| 64 | 59.60 | 0.08 | 0.67 | 1.11 | 16.91 | 50.60 | 54.22 | 57.17 | 59.60 | 62.06 | 65.14 | 69.06 |
| 65 | 59.72 | 0.08 | 0.66 | 1.11 | 17.16 | 50.73 | 54.35 | 57.30 | 59.72 | 62.18 | 65.27 | 69.20 |
| 66 | 59.85 | 0.08 | 0.64 | 1.11 | 17.41 | 50.87 | 54.48 | 57.42 | 59.85 | 62.31 | 65.40 | 69.34 |
| 67 | 59.97 | 0.08 | 0.62 | 1.11 | 17.67 | 51.00 | 54.60 | 57.54 | 59.97 | 62.43 | 65.52 | 69.48 |
| 68 | 60.09 | 0.08 | 0.60 | 1.11 | 17.92 | 51.13 | 54.72 | 57.66 | 60.09 | 62.56 | 65.65 | 69.62 |
| 69 | 60.21 | 0.08 | 0.58 | 1.11 | 18.17 | 51.26 | 54.85 | 57.78 | 60.21 | 62.68 | 65.78 | 69.76 |
| 70 | 60.33 | 0.08 | 0.56 | 1.11 | 18.42 | 51.38 | 54.97 | 57.90 | 60.33 | 62.80 | 65.91 | 69.90 |
| 71 | 60.45 | 0.08 | 0.54 | 1.11 | 18.68 | 51.51 | 55.09 | 58.02 | 60.45 | 62.92 | 66.04 | 70.05 |
| 72 | 60.57 | 0.08 | 0.51 | 1.11 | 18.93 | 51.63 | 55.20 | 58.14 | 60.57 | 63.05 | 66.17 | 70.19 |
| 73 | 60.69 | 0.08 | 0.49 | 1.11 | 19.18 | 51.75 | 55.32 | 58.25 | 60.69 | 63.17 | 66.30 | 70.34 |
| 74 | 60.80 | 0.08 | 0.47 | 1.11 | 19.43 | 51.87 | 55.43 | 58.37 | 60.80 | 63.29 | 66.43 | 70.49 |
| 75 | 60.92 | 0.08 | 0.44 | 1.11 | 19.69 | 51.99 | 55.55 | 58.48 | 60.92 | 63.41 | 66.56 | 70.64 |
| 76 | 61.03 | 0.08 | 0.42 | 1.11 | 19.94 | 52.11 | 55.66 | 58.59 | 61.03 | 63.53 | 66.69 | 70.79 |
| 77 | 61.15 | 0.08 | 0.39 | 1.11 | 20.19 | 52.23 | 55.77 | 58.70 | 61.15 | 63.65 | 66.83 | 70.94 |
| 78 | 61.26 | 0.08 | 0.37 | 1.11 | 20.44 | 52.34 | 55.88 | 58.81 | 61.26 | 63.77 | 66.96 | 71.09 |
| 79 | 61.37 | 0.08 | 0.34 | 1.11 | 20.70 | 52.45 | 55.99 | 58.92 | 61.37 | 63.89 | 67.09 | 71.24 |
| 80 | 61.49 | 0.08 | 0.31 | 1.11 | 20.95 | 52.56 | 56.09 | 59.03 | 61.49 | 64.01 | 67.22 | 71.40 |
| 81 | 61.60 | 0.08 | 0.29 | 1.11 | 21.20 | 52.67 | 56.20 | 59.14 | 61.60 | 64.13 | 67.35 | 71.55 |
| 82 | 61.71 | 0.08 | 0.26 | 1.11 | 21.45 | 52.78 | 56.30 | 59.24 | 61.71 | 64.25 | 67.48 | 71.71 |
| 83 | 61.82 | 0.08 | 0.23 | 1.11 | 21.71 | 52.88 | 56.41 | 59.35 | 61.82 | 64.36 | 67.62 | 71.87 |
| 84 | 61.93 | 0.08 | 0.21 | 1.11 | 21.96 | 52.98 | 56.51 | 59.45 | 61.93 | 64.48 | 67.75 | 72.03 |
| 85 | 62.03 | 0.08 | 0.18 | 1.11 | 22.21 | 53.08 | 56.61 | 59.56 | 62.03 | 64.60 | 67.88 | 72.19 |
| 86 | 62.14 | 0.08 | 0.15 | 1.11 | 22.46 | 53.18 | 56.71 | 59.66 | 62.14 | 64.72 | 68.02 | 72.35 |
| 87 | 62.25 | 0.08 | 0.12 | 1.11 | 22.72 | 53.28 | 56.80 | 59.76 | 62.25 | 64.83 | 68.15 | 72.51 |
| 88 | 62.36 | 0.08 | 0.10 | 1.11 | 22.97 | 53.38 | 56.90 | 59.86 | 62.36 | 64.95 | 68.28 | 72.68 |
| 89 | 62.46 | 0.08 | 0.07 | 1.11 | 23.22 | 53.47 | 56.99 | 59.96 | 62.46 | 65.07 | 68.42 | 72.84 |
| 90 | 62.57 | 0.08 | 0.04 | 1.11 | 23.47 | 53.57 | 57.09 | 60.06 | 62.57 | 65.18 | 68.55 | 73.01 |
| 91 | 62.68 | 0.08 | 0.02 | 1.11 | 23.73 | 53.66 | 57.18 | 60.16 | 62.68 | 65.30 | 68.69 | 73.18 |
| 92 | 62.78 | 0.08 | -0.01 | 1.11 | 23.98 | 53.75 | 57.27 | 60.25 | 62.78 | 65.41 | 68.82 | 73.34 |
| 93 | 62.89 | 0.08 | -0.04 | 1.11 | 24.23 | 53.84 | 57.37 | 60.35 | 62.89 | 65.53 | 68.96 | 73.51 |
| 94 | 62.99 | 0.08 | -0.06 | 1.11 | 24.48 | 53.93 | 57.46 | 60.45 | 62.99 | 65.65 | 69.09 | 73.68 |
| 95 | 63.09 | 0.08 | -0.09 | 1.11 | 24.74 | 54.02 | 57.55 | 60.54 | 63.09 | 65.76 | 69.23 | 73.85 |
| 96 | 63.20 | 0.08 | -0.11 | 1.11 | 24.99 | 54.11 | 57.64 | 60.64 | 63.20 | 65.88 | 69.36 | 74.02 |
| 97 | 63.30 | 0.08 | -0.14 | 1.11 | 25.24 | 54.20 | 57.73 | 60.74 | 63.30 | 65.99 | 69.50 | 74.19 |
| 98 | 63.41 | 0.08 | -0.17 | 1.11 | 25.49 | 54.29 | 57.82 | 60.83 | 63.41 | 66.11 | 69.64 | 74.37 |
| 99 | 63.51 | 0.08 | -0.19 | 1.11 | 25.75 | 54.38 | 57.91 | 60.93 | 63.51 | 66.23 | 69.77 | 74.54 |
| 100 | 63.62 | 0.08 | -0.22 | 1.11 | 26.00 | 54.46 | 58.00 | 61.03 | 63.62 | 66.34 | 69.91 | 74.71 |

**x**: is the age of child in weeks; C: Percentile

Table S10 Presentation of meu, sigma, lambda for each age and tau for H/A males curve with percentiles **(BCPE, ps, df (L=0, M=1, S=0))**

|  | $\boldsymbol{\mu}$ | $\boldsymbol{\sigma}$ | $\boldsymbol{\lambda}$ | $\boldsymbol{\tau}$ | x | C3 | C10 | C25 | C50 | C75 | C90 | C97 |
| --- | --- | --- | --- | --- | --- | --- | --- | --- | --- | --- | --- | --- |
| 1 | 49.48 | 0.09 | 1.23 | 1.19 | 1.00 | 40.41 | 44.07 | 47.03 | 49.48 | 51.91 | 54.76 | 58.19 |
| 2 | 49.69 | 0.09 | 1.24 | 1.19 | 1.25 | 40.59 | 44.27 | 47.23 | 49.69 | 52.12 | 54.97 | 58.40 |
| 3 | 49.90 | 0.09 | 1.25 | 1.19 | 1.51 | 40.78 | 44.46 | 47.43 | 49.90 | 52.33 | 55.18 | 58.61 |
| 4 | 50.10 | 0.09 | 1.26 | 1.19 | 1.76 | 40.97 | 44.66 | 47.64 | 50.10 | 52.54 | 55.39 | 58.82 |
| 5 | 50.31 | 0.09 | 1.27 | 1.19 | 2.01 | 41.15 | 44.86 | 47.84 | 50.31 | 52.75 | 55.60 | 59.03 |
| 6 | 50.51 | 0.09 | 1.28 | 1.19 | 2.26 | 41.34 | 45.05 | 48.04 | 50.51 | 52.95 | 55.81 | 59.24 |
| 7 | 50.72 | 0.09 | 1.29 | 1.19 | 2.52 | 41.53 | 45.25 | 48.24 | 50.72 | 53.16 | 56.02 | 59.45 |
| 8 | 50.92 | 0.09 | 1.30 | 1.19 | 2.77 | 41.72 | 45.45 | 48.44 | 50.92 | 53.37 | 56.23 | 59.66 |
| 9 | 51.13 | 0.09 | 1.31 | 1.19 | 3.02 | 41.90 | 45.64 | 48.65 | 51.13 | 53.58 | 56.44 | 59.87 |
| 10 | 51.33 | 0.09 | 1.32 | 1.19 | 3.27 | 42.09 | 45.84 | 48.85 | 51.33 | 53.78 | 56.65 | 60.08 |
| 11 | 51.54 | 0.09 | 1.33 | 1.19 | 3.53 | 42.28 | 46.03 | 49.05 | 51.54 | 53.99 | 56.86 | 60.29 |
| 12 | 51.74 | 0.09 | 1.33 | 1.19 | 3.78 | 42.46 | 46.23 | 49.25 | 51.74 | 54.20 | 57.06 | 60.49 |
| 13 | 51.95 | 0.09 | 1.34 | 1.19 | 4.03 | 42.65 | 46.42 | 49.45 | 51.95 | 54.40 | 57.27 | 60.70 |
| 14 | 52.15 | 0.09 | 1.35 | 1.19 | 4.28 | 42.83 | 46.62 | 49.65 | 52.15 | 54.61 | 57.48 | 60.91 |
| 15 | 52.35 | 0.09 | 1.36 | 1.19 | 4.54 | 43.02 | 46.81 | 49.85 | 52.35 | 54.81 | 57.68 | 61.11 |
| 16 | 52.55 | 0.09 | 1.37 | 1.19 | 4.79 | 43.20 | 47.01 | 50.05 | 52.55 | 55.02 | 57.89 | 61.32 |
| 17 | 52.75 | 0.09 | 1.38 | 1.19 | 5.04 | 43.39 | 47.20 | 50.24 | 52.75 | 55.22 | 58.09 | 61.52 |
| 18 | 52.95 | 0.09 | 1.39 | 1.19 | 5.29 | 43.57 | 47.39 | 50.44 | 52.95 | 55.42 | 58.29 | 61.72 |
| 19 | 53.15 | 0.09 | 1.40 | 1.19 | 5.55 | 43.76 | 47.59 | 50.64 | 53.15 | 55.62 | 58.50 | 61.93 |
| 20 | 53.35 | 0.09 | 1.41 | 1.19 | 5.80 | 43.94 | 47.78 | 50.83 | 53.35 | 55.82 | 58.70 | 62.13 |
| 21 | 53.55 | 0.09 | 1.42 | 1.19 | 6.05 | 44.12 | 47.97 | 51.03 | 53.55 | 56.02 | 58.90 | 62.33 |
| 22 | 53.75 | 0.09 | 1.43 | 1.19 | 6.30 | 44.31 | 48.16 | 51.22 | 53.75 | 56.22 | 59.10 | 62.52 |
| 23 | 53.94 | 0.09 | 1.44 | 1.19 | 6.56 | 44.49 | 48.35 | 51.42 | 53.94 | 56.42 | 59.30 | 62.72 |
| 24 | 54.14 | 0.09 | 1.44 | 1.19 | 6.81 | 44.67 | 48.53 | 51.61 | 54.14 | 56.61 | 59.49 | 62.92 |
| 25 | 54.33 | 0.08 | 1.45 | 1.19 | 7.06 | 44.85 | 48.72 | 51.80 | 54.33 | 56.81 | 59.69 | 63.11 |
| 26 | 54.52 | 0.08 | 1.46 | 1.19 | 7.31 | 45.03 | 48.91 | 51.99 | 54.52 | 57.00 | 59.88 | 63.31 |
| 27 | 54.72 | 0.08 | 1.47 | 1.19 | 7.57 | 45.21 | 49.10 | 52.18 | 54.72 | 57.20 | 60.08 | 63.50 |
| 28 | 54.91 | 0.08 | 1.48 | 1.19 | 7.82 | 45.38 | 49.28 | 52.37 | 54.91 | 57.39 | 60.27 | 63.69 |
| 29 | 55.10 | 0.08 | 1.49 | 1.19 | 8.07 | 45.56 | 49.46 | 52.56 | 55.10 | 57.58 | 60.46 | 63.88 |
| 30 | 55.29 | 0.08 | 1.50 | 1.19 | 8.32 | 45.74 | 49.65 | 52.75 | 55.29 | 57.77 | 60.65 | 64.07 |
| 31 | 55.47 | 0.08 | 1.51 | 1.19 | 8.58 | 45.91 | 49.83 | 52.93 | 55.47 | 57.96 | 60.84 | 64.26 |
| 32 | 55.66 | 0.08 | 1.52 | 1.19 | 8.83 | 46.09 | 50.01 | 53.12 | 55.66 | 58.14 | 61.03 | 64.44 |
| 33 | 55.85 | 0.08 | 1.53 | 1.19 | 9.08 | 46.26 | 50.19 | 53.30 | 55.85 | 58.33 | 61.21 | 64.63 |
| 34 | 56.03 | 0.08 | 1.54 | 1.19 | 9.33 | 46.44 | 50.37 | 53.48 | 56.03 | 58.52 | 61.40 | 64.81 |
| 35 | 56.21 | 0.08 | 1.55 | 1.19 | 9.59 | 46.61 | 50.55 | 53.66 | 56.21 | 58.70 | 61.58 | 64.99 |
| 36 | 56.39 | 0.08 | 1.55 | 1.19 | 9.84 | 46.78 | 50.73 | 53.84 | 56.39 | 58.88 | 61.76 | 65.17 |
| 37 | 56.57 | 0.08 | 1.56 | 1.19 | 10.09 | 46.95 | 50.90 | 54.02 | 56.57 | 59.06 | 61.94 | 65.35 |
| 38 | 56.75 | 0.08 | 1.57 | 1.19 | 10.34 | 47.12 | 51.08 | 54.20 | 56.75 | 59.24 | 62.12 | 65.52 |
| 39 | 56.93 | 0.08 | 1.58 | 1.19 | 10.60 | 47.29 | 51.25 | 54.38 | 56.93 | 59.42 | 62.29 | 65.70 |
| 40 | 57.10 | 0.08 | 1.59 | 1.19 | 10.85 | 47.45 | 51.42 | 54.55 | 57.10 | 59.59 | 62.47 | 65.87 |
| 41 | 57.28 | 0.08 | 1.60 | 1.19 | 11.10 | 47.62 | 51.59 | 54.72 | 57.28 | 59.77 | 62.64 | 66.04 |
| 42 | 57.45 | 0.08 | 1.61 | 1.19 | 11.35 | 47.78 | 51.76 | 54.90 | 57.45 | 59.94 | 62.81 | 66.21 |
| 43 | 57.62 | 0.08 | 1.62 | 1.19 | 11.61 | 47.95 | 51.93 | 55.07 | 57.62 | 60.11 | 62.98 | 66.38 |
| 44 | 57.79 | 0.08 | 1.63 | 1.19 | 11.86 | 48.11 | 52.10 | 55.24 | 57.79 | 60.28 | 63.15 | 66.55 |
| 45 | 57.96 | 0.08 | 1.64 | 1.19 | 12.11 | 48.27 | 52.26 | 55.40 | 57.96 | 60.45 | 63.32 | 66.71 |
| 46 | 58.13 | 0.08 | 1.65 | 1.19 | 12.36 | 48.43 | 52.43 | 55.57 | 58.13 | 60.61 | 63.49 | 66.87 |
| 47 | 58.29 | 0.08 | 1.66 | 1.19 | 12.62 | 48.59 | 52.59 | 55.73 | 58.29 | 60.78 | 63.65 | 67.03 |
| 48 | 58.46 | 0.08 | 1.67 | 1.19 | 12.87 | 48.75 | 52.75 | 55.90 | 58.46 | 60.94 | 63.81 | 67.19 |
| 49 | 58.62 | 0.08 | 1.67 | 1.19 | 13.12 | 48.90 | 52.91 | 56.06 | 58.62 | 61.10 | 63.97 | 67.35 |
| 50 | 58.78 | 0.08 | 1.68 | 1.19 | 13.37 | 49.06 | 53.07 | 56.22 | 58.78 | 61.26 | 64.13 | 67.50 |
| 51 | 58.94 | 0.08 | 1.69 | 1.19 | 13.63 | 49.21 | 53.23 | 56.38 | 58.94 | 61.42 | 64.28 | 67.66 |
| 52 | 59.09 | 0.08 | 1.70 | 1.19 | 13.88 | 49.37 | 53.39 | 56.54 | 59.09 | 61.58 | 64.44 | 67.81 |
| 53 | 59.25 | 0.08 | 1.71 | 1.19 | 14.13 | 49.52 | 53.54 | 56.69 | 59.25 | 61.73 | 64.59 | 67.96 |
| 54 | 59.40 | 0.08 | 1.72 | 1.19 | 14.38 | 49.67 | 53.69 | 56.84 | 59.40 | 61.88 | 64.74 | 68.10 |
| 55 | 59.56 | 0.08 | 1.73 | 1.19 | 14.64 | 49.82 | 53.85 | 57.00 | 59.56 | 62.04 | 64.89 | 68.25 |
| 56 | 59.71 | 0.08 | 1.74 | 1.19 | 14.89 | 49.96 | 54.00 | 57.15 | 59.71 | 62.19 | 65.04 | 68.39 |
| 57 | 59.86 | 0.08 | 1.75 | 1.19 | 15.14 | 50.11 | 54.15 | 57.30 | 59.86 | 62.33 | 65.18 | 68.53 |
| 58 | 60.00 | 0.08 | 1.76 | 1.19 | 15.39 | 50.26 | 54.29 | 57.45 | 60.00 | 62.48 | 65.33 | 68.67 |
| 59 | 60.15 | 0.08 | 1.77 | 1.19 | 15.65 | 50.40 | 54.44 | 57.59 | 60.15 | 62.62 | 65.47 | 68.81 |
| 60 | 60.29 | 0.08 | 1.78 | 1.19 | 15.90 | 50.54 | 54.59 | 57.74 | 60.29 | 62.77 | 65.61 | 68.95 |
| 61 | 60.44 | 0.08 | 1.78 | 1.19 | 16.15 | 50.69 | 54.73 | 57.88 | 60.44 | 62.91 | 65.75 | 69.08 |
| 62 | 60.58 | 0.08 | 1.79 | 1.19 | 16.40 | 50.83 | 54.87 | 58.03 | 60.58 | 63.05 | 65.89 | 69.22 |
| 63 | 60.72 | 0.08 | 1.80 | 1.19 | 16.66 | 50.97 | 55.01 | 58.17 | 60.72 | 63.19 | 66.02 | 69.35 |
| 64 | 60.86 | 0.08 | 1.81 | 1.19 | 16.91 | 51.11 | 55.15 | 58.31 | 60.86 | 63.32 | 66.16 | 69.48 |
| 65 | 61.00 | 0.08 | 1.82 | 1.19 | 17.16 | 51.24 | 55.29 | 58.45 | 61.00 | 63.46 | 66.29 | 69.61 |
| 66 | 61.13 | 0.08 | 1.83 | 1.19 | 17.41 | 51.38 | 55.43 | 58.58 | 61.13 | 63.60 | 66.42 | 69.73 |
| 67 | 61.27 | 0.08 | 1.84 | 1.19 | 17.67 | 51.52 | 55.57 | 58.72 | 61.27 | 63.73 | 66.55 | 69.86 |
| 68 | 61.40 | 0.08 | 1.85 | 1.19 | 17.92 | 51.65 | 55.71 | 58.86 | 61.40 | 63.86 | 66.68 | 69.99 |
| 69 | 61.54 | 0.07 | 1.86 | 1.19 | 18.17 | 51.79 | 55.84 | 58.99 | 61.54 | 63.99 | 66.81 | 70.11 |
| 70 | 61.67 | 0.07 | 1.87 | 1.19 | 18.42 | 51.92 | 55.98 | 59.13 | 61.67 | 64.12 | 66.94 | 70.23 |
| 71 | 61.80 | 0.07 | 1.88 | 1.19 | 18.68 | 52.05 | 56.11 | 59.26 | 61.80 | 64.25 | 67.07 | 70.35 |
| 72 | 61.93 | 0.07 | 1.89 | 1.19 | 18.93 | 52.19 | 56.24 | 59.39 | 61.93 | 64.38 | 67.19 | 70.48 |
| 73 | 62.06 | 0.07 | 1.89 | 1.19 | 19.18 | 52.32 | 56.38 | 59.52 | 62.06 | 64.51 | 67.32 | 70.60 |
| 74 | 62.19 | 0.07 | 1.90 | 1.19 | 19.43 | 52.45 | 56.51 | 59.66 | 62.19 | 64.64 | 67.44 | 70.72 |
| 75 | 62.32 | 0.07 | 1.91 | 1.19 | 19.69 | 52.58 | 56.64 | 59.79 | 62.32 | 64.77 | 67.56 | 70.83 |
| 76 | 62.45 | 0.07 | 1.92 | 1.19 | 19.94 | 52.71 | 56.77 | 59.92 | 62.45 | 64.89 | 67.69 | 70.95 |
| 77 | 62.58 | 0.07 | 1.93 | 1.19 | 20.19 | 52.84 | 56.90 | 60.05 | 62.58 | 65.02 | 67.81 | 71.07 |
| 78 | 62.70 | 0.07 | 1.94 | 1.19 | 20.44 | 52.97 | 57.03 | 60.17 | 62.71 | 65.14 | 67.93 | 71.19 |
| 79 | 62.83 | 0.07 | 1.95 | 1.19 | 20.70 | 53.10 | 57.16 | 60.30 | 62.83 | 65.27 | 68.05 | 71.30 |
| 80 | 62.96 | 0.07 | 1.96 | 1.19 | 20.95 | 53.23 | 57.29 | 60.43 | 62.96 | 65.39 | 68.17 | 71.42 |
| 81 | 63.08 | 0.07 | 1.97 | 1.19 | 21.20 | 53.36 | 57.42 | 60.56 | 63.08 | 65.51 | 68.29 | 71.53 |
| 82 | 63.21 | 0.07 | 1.98 | 1.19 | 21.45 | 53.49 | 57.55 | 60.69 | 63.21 | 65.64 | 68.41 | 71.65 |
| 83 | 63.33 | 0.07 | 1.99 | 1.19 | 21.71 | 53.62 | 57.68 | 60.81 | 63.33 | 65.76 | 68.53 | 71.76 |
| 84 | 63.46 | 0.07 | 2.00 | 1.19 | 21.96 | 53.75 | 57.81 | 60.94 | 63.46 | 65.88 | 68.65 | 71.88 |
| 85 | 63.58 | 0.07 | 2.01 | 1.19 | 22.21 | 53.87 | 57.93 | 61.07 | 63.58 | 66.00 | 68.77 | 71.99 |
| 86 | 63.71 | 0.07 | 2.01 | 1.19 | 22.46 | 54.00 | 58.06 | 61.19 | 63.71 | 66.13 | 68.89 | 72.10 |
| 87 | 63.83 | 0.07 | 2.02 | 1.19 | 22.72 | 54.13 | 58.19 | 61.32 | 63.83 | 66.25 | 69.00 | 72.22 |
| 88 | 63.96 | 0.07 | 2.03 | 1.19 | 22.97 | 54.26 | 58.32 | 61.44 | 63.96 | 66.37 | 69.12 | 72.33 |
| 89 | 64.08 | 0.07 | 2.04 | 1.19 | 23.22 | 54.39 | 58.44 | 61.57 | 64.08 | 66.49 | 69.24 | 72.44 |
| 90 | 64.20 | 0.07 | 2.05 | 1.19 | 23.47 | 54.51 | 58.57 | 61.70 | 64.20 | 66.61 | 69.36 | 72.55 |
| 91 | 64.33 | 0.07 | 2.06 | 1.19 | 23.73 | 54.64 | 58.70 | 61.82 | 64.33 | 66.73 | 69.47 | 72.67 |
| 92 | 64.45 | 0.07 | 2.07 | 1.19 | 23.98 | 54.77 | 58.83 | 61.95 | 64.45 | 66.85 | 69.59 | 72.78 |
| 93 | 64.57 | 0.07 | 2.08 | 1.19 | 24.23 | 54.90 | 58.95 | 62.07 | 64.57 | 66.97 | 69.71 | 72.89 |
| 94 | 64.70 | 0.07 | 2.09 | 1.19 | 24.48 | 55.02 | 59.08 | 62.20 | 64.70 | 67.09 | 69.83 | 73.00 |
| 95 | 64.82 | 0.07 | 2.10 | 1.19 | 24.74 | 55.15 | 59.21 | 62.32 | 64.82 | 67.21 | 69.94 | 73.12 |
| 96 | 64.94 | 0.07 | 2.11 | 1.19 | 24.99 | 55.28 | 59.33 | 62.45 | 64.94 | 67.34 | 70.06 | 73.23 |
| 97 | 65.07 | 0.07 | 2.12 | 1.19 | 25.24 | 55.41 | 59.46 | 62.57 | 65.07 | 67.46 | 70.18 | 73.34 |
| 98 | 65.19 | 0.07 | 2.12 | 1.19 | 25.49 | 55.54 | 59.59 | 62.70 | 65.19 | 67.58 | 70.29 | 73.45 |
| 99 | 65.31 | 0.07 | 2.13 | 1.19 | 25.75 | 55.66 | 59.72 | 62.82 | 65.31 | 67.70 | 70.41 | 73.56 |
| 100 | 65.44 | 0.07 | 2.14 | 1.19 | 26.00 | 55.79 | 59.84 | 62.95 | 65.44 | 67.82 | 70.53 | 73.68 |

**x**: is the age of child in weeks; C: Percentile

Table S11 Presentation of meu, sigma, lambda for each age and tau for W/A females curve with percentiles **(BCPE, ps, df (L=0, M=1, S=0))**

|  | $\boldsymbol{\mu}$ | $\boldsymbol{\sigma}$ | $\boldsymbol{\lambda}$ | $\boldsymbol{\tau}$ | **x** | **C3** | **C10** | **C25** | **C50** | **C75** | **C90** | **C97** |
| --- | --- | --- | --- | --- | --- | --- | --- | --- | --- | --- | --- | --- |
| **1** | 3.12 | 0.22 | -0.09 | 1.45 | 1.00 | 2.05 | 2.38 | 2.73 | 3.12 | 3.56 | 4.10 | 4.82 |
| **2** | 3.16 | 0.22 | -0.09 | 1.45 | 1.25 | 2.08 | 2.42 | 2.77 | 3.16 | 3.60 | 4.15 | 4.88 |
| **3** | 3.20 | 0.22 | -0.08 | 1.45 | 1.51 | 2.10 | 2.45 | 2.81 | 3.20 | 3.65 | 4.21 | 4.94 |
| **4** | 3.24 | 0.22 | -0.07 | 1.45 | 1.76 | 2.13 | 2.48 | 2.84 | 3.24 | 3.70 | 4.26 | 4.99 |
| **5** | 3.28 | 0.22 | -0.06 | 1.45 | 2.01 | 2.15 | 2.51 | 2.88 | 3.28 | 3.74 | 4.31 | 5.05 |
| **6** | 3.32 | 0.22 | -0.05 | 1.45 | 2.26 | 2.18 | 2.54 | 2.92 | 3.32 | 3.79 | 4.36 | 5.11 |
| **7** | 3.36 | 0.22 | -0.05 | 1.45 | 2.52 | 2.21 | 2.57 | 2.95 | 3.36 | 3.83 | 4.41 | 5.17 |
| **8** | 3.40 | 0.22 | -0.04 | 1.45 | 2.77 | 2.23 | 2.60 | 2.99 | 3.40 | 3.88 | 4.46 | 5.22 |
| **9** | 3.44 | 0.22 | -0.03 | 1.45 | 3.02 | 2.26 | 2.64 | 3.02 | 3.44 | 3.93 | 4.51 | 5.28 |
| **10** | 3.49 | 0.22 | -0.02 | 1.45 | 3.27 | 2.29 | 2.67 | 3.06 | 3.49 | 3.97 | 4.56 | 5.34 |
| **11** | 3.53 | 0.22 | -0.02 | 1.45 | 3.53 | 2.31 | 2.70 | 3.10 | 3.53 | 4.02 | 4.61 | 5.39 |
| **12** | 3.57 | 0.22 | -0.01 | 1.45 | 3.78 | 2.34 | 2.73 | 3.13 | 3.57 | 4.06 | 4.67 | 5.45 |
| **13** | 3.61 | 0.22 | 0.00 | 1.45 | 4.03 | 2.36 | 2.76 | 3.17 | 3.61 | 4.11 | 4.72 | 5.51 |
| **14** | 3.65 | 0.22 | 0.01 | 1.45 | 4.28 | 2.39 | 2.79 | 3.20 | 3.65 | 4.15 | 4.77 | 5.56 |
| **15** | 3.69 | 0.22 | 0.02 | 1.45 | 4.54 | 2.42 | 2.82 | 3.24 | 3.69 | 4.20 | 4.82 | 5.62 |
| **16** | 3.73 | 0.22 | 0.02 | 1.45 | 4.79 | 2.44 | 2.85 | 3.28 | 3.73 | 4.25 | 4.87 | 5.67 |
| **17** | 3.77 | 0.22 | 0.03 | 1.45 | 5.04 | 2.47 | 2.88 | 3.31 | 3.77 | 4.29 | 4.92 | 5.73 |
| **18** | 3.81 | 0.22 | 0.04 | 1.45 | 5.29 | 2.49 | 2.92 | 3.35 | 3.81 | 4.34 | 4.97 | 5.78 |
| **19** | 3.85 | 0.22 | 0.05 | 1.45 | 5.55 | 2.52 | 2.95 | 3.38 | 3.85 | 4.38 | 5.02 | 5.84 |
| **20** | 3.89 | 0.22 | 0.05 | 1.45 | 5.80 | 2.55 | 2.98 | 3.42 | 3.89 | 4.43 | 5.07 | 5.89 |
| **21** | 3.93 | 0.22 | 0.06 | 1.45 | 6.05 | 2.57 | 3.01 | 3.45 | 3.93 | 4.47 | 5.12 | 5.95 |
| **22** | 3.97 | 0.22 | 0.07 | 1.45 | 6.30 | 2.60 | 3.04 | 3.49 | 3.97 | 4.51 | 5.17 | 6.00 |
| **23** | 4.01 | 0.22 | 0.08 | 1.45 | 6.56 | 2.62 | 3.07 | 3.52 | 4.01 | 4.56 | 5.21 | 6.05 |
| **24** | 4.05 | 0.22 | 0.09 | 1.45 | 6.81 | 2.65 | 3.10 | 3.56 | 4.05 | 4.60 | 5.26 | 6.11 |
| **25** | 4.09 | 0.21 | 0.09 | 1.45 | 7.06 | 2.67 | 3.13 | 3.59 | 4.09 | 4.65 | 5.31 | 6.16 |
| **26** | 4.13 | 0.21 | 0.10 | 1.45 | 7.31 | 2.70 | 3.16 | 3.63 | 4.13 | 4.69 | 5.36 | 6.21 |
| **27** | 4.17 | 0.21 | 0.11 | 1.45 | 7.57 | 2.72 | 3.19 | 3.66 | 4.17 | 4.73 | 5.41 | 6.26 |
| **28** | 4.21 | 0.21 | 0.12 | 1.45 | 7.82 | 2.75 | 3.22 | 3.70 | 4.21 | 4.78 | 5.45 | 6.31 |
| **29** | 4.24 | 0.21 | 0.12 | 1.45 | 8.07 | 2.77 | 3.25 | 3.73 | 4.24 | 4.82 | 5.50 | 6.36 |
| **30** | 4.28 | 0.21 | 0.13 | 1.45 | 8.32 | 2.79 | 3.28 | 3.76 | 4.28 | 4.86 | 5.55 | 6.42 |
| **31** | 4.32 | 0.21 | 0.14 | 1.45 | 8.58 | 2.82 | 3.30 | 3.80 | 4.32 | 4.90 | 5.59 | 6.47 |
| **32** | 4.36 | 0.21 | 0.15 | 1.45 | 8.83 | 2.84 | 3.33 | 3.83 | 4.36 | 4.94 | 5.64 | 6.52 |
| **33** | 4.40 | 0.21 | 0.16 | 1.45 | 9.08 | 2.87 | 3.36 | 3.86 | 4.40 | 4.99 | 5.68 | 6.56 |
| **34** | 4.43 | 0.21 | 0.16 | 1.45 | 9.33 | 2.89 | 3.39 | 3.90 | 4.43 | 5.03 | 5.73 | 6.61 |
| **35** | 4.47 | 0.21 | 0.17 | 1.45 | 9.59 | 2.91 | 3.42 | 3.93 | 4.47 | 5.07 | 5.78 | 6.66 |
| **36** | 4.51 | 0.21 | 0.18 | 1.45 | 9.84 | 2.94 | 3.45 | 3.96 | 4.51 | 5.11 | 5.82 | 6.71 |
| **37** | 4.54 | 0.21 | 0.19 | 1.45 | 10.09 | 2.96 | 3.48 | 4.00 | 4.54 | 5.15 | 5.86 | 6.76 |
| **38** | 4.58 | 0.21 | 0.19 | 1.45 | 10.34 | 2.98 | 3.50 | 4.03 | 4.58 | 5.19 | 5.91 | 6.81 |
| **39** | 4.62 | 0.21 | 0.20 | 1.45 | 10.60 | 3.00 | 3.53 | 4.06 | 4.62 | 5.23 | 5.95 | 6.85 |
| **40** | 4.65 | 0.21 | 0.21 | 1.45 | 10.85 | 3.03 | 3.56 | 4.09 | 4.65 | 5.27 | 6.00 | 6.90 |
| **41** | 4.69 | 0.21 | 0.22 | 1.45 | 11.10 | 3.05 | 3.59 | 4.12 | 4.69 | 5.31 | 6.04 | 6.95 |
| **42** | 4.72 | 0.21 | 0.23 | 1.45 | 11.35 | 3.07 | 3.61 | 4.16 | 4.72 | 5.35 | 6.08 | 6.99 |
| **43** | 4.76 | 0.21 | 0.23 | 1.45 | 11.61 | 3.09 | 3.64 | 4.19 | 4.76 | 5.39 | 6.13 | 7.04 |
| **44** | 4.79 | 0.21 | 0.24 | 1.45 | 11.86 | 3.12 | 3.67 | 4.22 | 4.79 | 5.43 | 6.17 | 7.08 |
| **45** | 4.83 | 0.21 | 0.25 | 1.45 | 12.11 | 3.14 | 3.69 | 4.25 | 4.83 | 5.47 | 6.21 | 7.13 |
| **46** | 4.86 | 0.21 | 0.26 | 1.45 | 12.36 | 3.16 | 3.72 | 4.28 | 4.86 | 5.51 | 6.25 | 7.17 |
| **47** | 4.90 | 0.21 | 0.26 | 1.45 | 12.62 | 3.18 | 3.75 | 4.31 | 4.90 | 5.54 | 6.29 | 7.22 |
| **48** | 4.93 | 0.21 | 0.27 | 1.45 | 12.87 | 3.20 | 3.77 | 4.34 | 4.93 | 5.58 | 6.33 | 7.26 |
| **49** | 4.97 | 0.21 | 0.28 | 1.45 | 13.12 | 3.22 | 3.80 | 4.37 | 4.97 | 5.62 | 6.37 | 7.31 |
| **50** | 5.00 | 0.21 | 0.29 | 1.45 | 13.37 | 3.24 | 3.83 | 4.40 | 5.00 | 5.66 | 6.42 | 7.35 |
| **51** | 5.04 | 0.21 | 0.30 | 1.45 | 13.63 | 3.27 | 3.85 | 4.43 | 5.04 | 5.69 | 6.46 | 7.39 |
| **52** | 5.07 | 0.21 | 0.30 | 1.45 | 13.88 | 3.29 | 3.88 | 4.46 | 5.07 | 5.73 | 6.50 | 7.43 |
| **53** | 5.10 | 0.21 | 0.31 | 1.45 | 14.13 | 3.31 | 3.90 | 4.49 | 5.10 | 5.77 | 6.54 | 7.48 |
| **54** | 5.14 | 0.21 | 0.32 | 1.45 | 14.38 | 3.33 | 3.93 | 4.52 | 5.14 | 5.80 | 6.57 | 7.52 |
| **55** | 5.17 | 0.21 | 0.33 | 1.45 | 14.64 | 3.35 | 3.95 | 4.55 | 5.17 | 5.84 | 6.61 | 7.56 |
| **56** | 5.20 | 0.21 | 0.33 | 1.45 | 14.89 | 3.37 | 3.98 | 4.58 | 5.20 | 5.88 | 6.65 | 7.60 |
| **57** | 5.23 | 0.21 | 0.34 | 1.45 | 15.14 | 3.39 | 4.00 | 4.61 | 5.23 | 5.91 | 6.69 | 7.64 |
| **58** | 5.27 | 0.21 | 0.35 | 1.45 | 15.39 | 3.41 | 4.03 | 4.64 | 5.27 | 5.95 | 6.73 | 7.68 |
| **59** | 5.30 | 0.21 | 0.36 | 1.45 | 15.65 | 3.43 | 4.05 | 4.67 | 5.30 | 5.98 | 6.77 | 7.72 |
| **60** | 5.33 | 0.21 | 0.37 | 1.45 | 15.90 | 3.45 | 4.08 | 4.70 | 5.33 | 6.02 | 6.80 | 7.76 |
| **61** | 5.36 | 0.21 | 0.37 | 1.45 | 16.15 | 3.47 | 4.10 | 4.72 | 5.36 | 6.05 | 6.84 | 7.80 |
| **62** | 5.39 | 0.21 | 0.38 | 1.45 | 16.40 | 3.49 | 4.13 | 4.75 | 5.39 | 6.09 | 6.88 | 7.84 |
| **63** | 5.42 | 0.21 | 0.39 | 1.45 | 16.66 | 3.51 | 4.15 | 4.78 | 5.42 | 6.12 | 6.92 | 7.88 |
| **64** | 5.46 | 0.21 | 0.40 | 1.45 | 16.91 | 3.52 | 4.17 | 4.81 | 5.46 | 6.15 | 6.95 | 7.92 |
| **65** | 5.49 | 0.21 | 0.40 | 1.45 | 17.16 | 3.54 | 4.20 | 4.83 | 5.49 | 6.19 | 6.99 | 7.95 |
| **66** | 5.52 | 0.21 | 0.41 | 1.45 | 17.41 | 3.56 | 4.22 | 4.86 | 5.52 | 6.22 | 7.02 | 7.99 |
| **67** | 5.55 | 0.21 | 0.42 | 1.45 | 17.67 | 3.58 | 4.24 | 4.89 | 5.55 | 6.26 | 7.06 | 8.03 |
| **68** | 5.58 | 0.21 | 0.43 | 1.45 | 17.92 | 3.60 | 4.27 | 4.92 | 5.58 | 6.29 | 7.10 | 8.07 |
| **69** | 5.61 | 0.21 | 0.44 | 1.45 | 18.17 | 3.62 | 4.29 | 4.94 | 5.61 | 6.32 | 7.13 | 8.10 |
| **70** | 5.64 | 0.21 | 0.44 | 1.45 | 18.42 | 3.64 | 4.31 | 4.97 | 5.64 | 6.35 | 7.17 | 8.14 |
| **71** | 5.67 | 0.21 | 0.45 | 1.45 | 18.68 | 3.65 | 4.33 | 5.00 | 5.67 | 6.39 | 7.20 | 8.18 |
| **72** | 5.70 | 0.20 | 0.46 | 1.45 | 18.93 | 3.67 | 4.36 | 5.02 | 5.70 | 6.42 | 7.23 | 8.21 |
| **73** | 5.73 | 0.20 | 0.47 | 1.45 | 19.18 | 3.69 | 4.38 | 5.05 | 5.73 | 6.45 | 7.27 | 8.25 |
| **74** | 5.76 | 0.20 | 0.47 | 1.45 | 19.43 | 3.71 | 4.40 | 5.08 | 5.76 | 6.48 | 7.30 | 8.28 |
| **75** | 5.79 | 0.20 | 0.48 | 1.45 | 19.69 | 3.72 | 4.42 | 5.10 | 5.79 | 6.51 | 7.34 | 8.32 |
| **76** | 5.81 | 0.20 | 0.49 | 1.45 | 19.94 | 3.74 | 4.45 | 5.13 | 5.81 | 6.55 | 7.37 | 8.35 |
| **77** | 5.84 | 0.20 | 0.50 | 1.45 | 20.19 | 3.76 | 4.47 | 5.15 | 5.84 | 6.58 | 7.40 | 8.39 |
| **78** | 5.87 | 0.20 | 0.51 | 1.45 | 20.44 | 3.78 | 4.49 | 5.18 | 5.87 | 6.61 | 7.44 | 8.42 |
| **79** | 5.90 | 0.20 | 0.51 | 1.45 | 20.70 | 3.79 | 4.51 | 5.20 | 5.90 | 6.64 | 7.47 | 8.46 |
| **80** | 5.93 | 0.20 | 0.52 | 1.45 | 20.95 | 3.81 | 4.53 | 5.23 | 5.93 | 6.67 | 7.50 | 8.49 |
| **81** | 5.96 | 0.20 | 0.53 | 1.45 | 21.20 | 3.83 | 4.56 | 5.26 | 5.96 | 6.70 | 7.53 | 8.52 |
| **82** | 5.99 | 0.20 | 0.54 | 1.45 | 21.45 | 3.85 | 4.58 | 5.28 | 5.99 | 6.73 | 7.57 | 8.56 |
| **83** | 6.01 | 0.20 | 0.54 | 1.45 | 21.71 | 3.86 | 4.60 | 5.31 | 6.01 | 6.76 | 7.60 | 8.59 |
| **84** | 6.04 | 0.20 | 0.55 | 1.45 | 21.96 | 3.88 | 4.62 | 5.33 | 6.04 | 6.79 | 7.63 | 8.62 |
| **85** | 6.07 | 0.20 | 0.56 | 1.45 | 22.21 | 3.90 | 4.64 | 5.36 | 6.07 | 6.82 | 7.66 | 8.66 |
| **86** | 6.10 | 0.20 | 0.57 | 1.45 | 22.46 | 3.91 | 4.66 | 5.38 | 6.10 | 6.85 | 7.70 | 8.69 |
| **87** | 6.13 | 0.20 | 0.58 | 1.45 | 22.72 | 3.93 | 4.68 | 5.41 | 6.13 | 6.88 | 7.73 | 8.72 |
| **88** | 6.15 | 0.20 | 0.58 | 1.45 | 22.97 | 3.95 | 4.71 | 5.43 | 6.15 | 6.91 | 7.76 | 8.76 |
| **89** | 6.18 | 0.20 | 0.59 | 1.45 | 23.22 | 3.96 | 4.73 | 5.46 | 6.18 | 6.94 | 7.79 | 8.79 |
| **90** | 6.21 | 0.20 | 0.60 | 1.45 | 23.47 | 3.98 | 4.75 | 5.48 | 6.21 | 6.97 | 7.82 | 8.82 |
| **91** | 6.24 | 0.20 | 0.61 | 1.45 | 23.73 | 3.99 | 4.77 | 5.50 | 6.24 | 7.00 | 7.85 | 8.85 |
| **92** | 6.26 | 0.20 | 0.61 | 1.45 | 23.98 | 4.01 | 4.79 | 5.53 | 6.26 | 7.03 | 7.88 | 8.88 |
| **93** | 6.29 | 0.20 | 0.62 | 1.45 | 24.23 | 4.03 | 4.81 | 5.55 | 6.29 | 7.06 | 7.92 | 8.92 |
| **94** | 6.32 | 0.20 | 0.63 | 1.45 | 24.48 | 4.04 | 4.83 | 5.58 | 6.32 | 7.09 | 7.95 | 8.95 |
| **95** | 6.34 | 0.20 | 0.64 | 1.45 | 24.74 | 4.06 | 4.85 | 5.60 | 6.34 | 7.12 | 7.98 | 8.98 |
| **96** | 6.37 | 0.20 | 0.65 | 1.45 | 24.99 | 4.07 | 4.87 | 5.63 | 6.37 | 7.15 | 8.01 | 9.01 |
| **97** | 6.40 | 0.20 | 0.65 | 1.45 | 25.24 | 4.09 | 4.89 | 5.65 | 6.40 | 7.18 | 8.04 | 9.04 |
| **98** | 6.43 | 0.20 | 0.66 | 1.45 | 25.49 | 4.11 | 4.91 | 5.68 | 6.43 | 7.21 | 8.07 | 9.07 |
| **99** | 6.45 | 0.20 | 0.67 | 1.45 | 25.75 | 4.12 | 4.94 | 5.70 | 6.45 | 7.24 | 8.10 | 9.11 |
| **100** | 6.48 | 0.20 | 0.68 | 1.45 | 26.00 | 4.14 | 4.96 | 5.72 | 6.48 | 7.27 | 8.13 | 9.14 |

**x**: is the age of child in weeks; C: Percentile

Table S12 Presentation of meu, sigma, lambda for each age and tau for W/A males curve with percentiles **(BCPE, ps, df (L=0, M=1, S=0))**

|  | $\boldsymbol{\mu}$ | $\boldsymbol{\sigma}$ | $\boldsymbol{\lambda}$ | $\boldsymbol{\tau}$ | x | C3 | C10 | C25 | C50 | C75 | C90 | C97 |
| --- | --- | --- | --- | --- | --- | --- | --- | --- | --- | --- | --- | --- |
| 1 | 3.19 | 0.23 | 0.05 | 1.66 | 1.00 | 2.05 | 2.40 | 2.76 | 3.19 | 3.68 | 4.24 | 4.91 |
| 2 | 3.24 | 0.23 | 0.06 | 1.66 | 1.25 | 2.08 | 2.43 | 2.80 | 3.24 | 3.74 | 4.30 | 4.97 |
| 3 | 3.29 | 0.23 | 0.08 | 1.66 | 1.51 | 2.11 | 2.47 | 2.85 | 3.29 | 3.79 | 4.35 | 5.03 |
| 4 | 3.33 | 0.23 | 0.09 | 1.66 | 1.76 | 2.14 | 2.50 | 2.89 | 3.33 | 3.84 | 4.41 | 5.09 |
| 5 | 3.38 | 0.23 | 0.10 | 1.66 | 2.01 | 2.17 | 2.54 | 2.93 | 3.38 | 3.89 | 4.46 | 5.15 |
| 6 | 3.42 | 0.22 | 0.11 | 1.66 | 2.26 | 2.20 | 2.57 | 2.97 | 3.42 | 3.94 | 4.52 | 5.21 |
| 7 | 3.47 | 0.22 | 0.13 | 1.66 | 2.52 | 2.23 | 2.61 | 3.01 | 3.47 | 3.99 | 4.57 | 5.27 |
| 8 | 3.52 | 0.22 | 0.14 | 1.66 | 2.77 | 2.26 | 2.64 | 3.05 | 3.52 | 4.04 | 4.63 | 5.33 |
| 9 | 3.56 | 0.22 | 0.15 | 1.66 | 3.02 | 2.29 | 2.68 | 3.09 | 3.56 | 4.10 | 4.69 | 5.39 |
| 10 | 3.61 | 0.22 | 0.16 | 1.66 | 3.27 | 2.32 | 2.71 | 3.13 | 3.61 | 4.15 | 4.74 | 5.45 |
| 11 | 3.66 | 0.22 | 0.18 | 1.66 | 3.53 | 2.35 | 2.75 | 3.17 | 3.66 | 4.20 | 4.80 | 5.51 |
| 12 | 3.70 | 0.22 | 0.19 | 1.66 | 3.78 | 2.38 | 2.78 | 3.21 | 3.70 | 4.25 | 4.85 | 5.56 |
| 13 | 3.75 | 0.22 | 0.20 | 1.66 | 4.03 | 2.41 | 2.82 | 3.25 | 3.75 | 4.30 | 4.91 | 5.62 |
| 14 | 3.79 | 0.22 | 0.22 | 1.66 | 4.28 | 2.44 | 2.85 | 3.29 | 3.79 | 4.35 | 4.96 | 5.68 |
| 15 | 3.84 | 0.22 | 0.23 | 1.66 | 4.54 | 2.47 | 2.89 | 3.33 | 3.84 | 4.40 | 5.01 | 5.74 |
| 16 | 3.88 | 0.22 | 0.24 | 1.66 | 4.79 | 2.50 | 2.92 | 3.37 | 3.88 | 4.45 | 5.07 | 5.79 |
| 17 | 3.93 | 0.22 | 0.25 | 1.66 | 5.04 | 2.52 | 2.96 | 3.41 | 3.93 | 4.50 | 5.12 | 5.85 |
| 18 | 3.98 | 0.22 | 0.27 | 1.66 | 5.29 | 2.55 | 2.99 | 3.45 | 3.98 | 4.55 | 5.18 | 5.91 |
| 19 | 4.02 | 0.22 | 0.28 | 1.66 | 5.55 | 2.58 | 3.03 | 3.50 | 4.02 | 4.60 | 5.23 | 5.96 |
| 20 | 4.07 | 0.22 | 0.29 | 1.66 | 5.80 | 2.61 | 3.06 | 3.54 | 4.07 | 4.65 | 5.28 | 6.02 |
| 21 | 4.11 | 0.22 | 0.30 | 1.66 | 6.05 | 2.64 | 3.10 | 3.58 | 4.11 | 4.70 | 5.33 | 6.07 |
| 22 | 4.16 | 0.22 | 0.32 | 1.66 | 6.30 | 2.67 | 3.13 | 3.62 | 4.16 | 4.75 | 5.39 | 6.13 |
| 23 | 4.20 | 0.21 | 0.33 | 1.66 | 6.56 | 2.70 | 3.17 | 3.66 | 4.20 | 4.80 | 5.44 | 6.18 |
| 24 | 4.24 | 0.21 | 0.34 | 1.66 | 6.81 | 2.73 | 3.20 | 3.69 | 4.24 | 4.85 | 5.49 | 6.24 |
| 25 | 4.29 | 0.21 | 0.35 | 1.66 | 7.06 | 2.75 | 3.23 | 3.73 | 4.29 | 4.90 | 5.54 | 6.29 |
| 26 | 4.33 | 0.21 | 0.37 | 1.66 | 7.31 | 2.78 | 3.27 | 3.77 | 4.33 | 4.94 | 5.60 | 6.34 |
| 27 | 4.38 | 0.21 | 0.38 | 1.66 | 7.57 | 2.81 | 3.30 | 3.81 | 4.38 | 4.99 | 5.65 | 6.40 |
| 28 | 4.42 | 0.21 | 0.39 | 1.66 | 7.82 | 2.84 | 3.34 | 3.85 | 4.42 | 5.04 | 5.70 | 6.45 |
| 29 | 4.47 | 0.21 | 0.40 | 1.66 | 8.07 | 2.87 | 3.37 | 3.89 | 4.47 | 5.09 | 5.75 | 6.50 |
| 30 | 4.51 | 0.21 | 0.42 | 1.66 | 8.32 | 2.89 | 3.40 | 3.93 | 4.51 | 5.13 | 5.80 | 6.55 |
| 31 | 4.55 | 0.21 | 0.43 | 1.66 | 8.58 | 2.92 | 3.44 | 3.97 | 4.55 | 5.18 | 5.85 | 6.61 |
| 32 | 4.60 | 0.21 | 0.44 | 1.66 | 8.83 | 2.95 | 3.47 | 4.01 | 4.60 | 5.23 | 5.90 | 6.66 |
| 33 | 4.64 | 0.21 | 0.46 | 1.66 | 9.08 | 2.98 | 3.50 | 4.05 | 4.64 | 5.28 | 5.95 | 6.71 |
| 34 | 4.68 | 0.21 | 0.47 | 1.66 | 9.33 | 3.00 | 3.54 | 4.08 | 4.68 | 5.32 | 6.00 | 6.76 |
| 35 | 4.72 | 0.21 | 0.48 | 1.66 | 9.59 | 3.03 | 3.57 | 4.12 | 4.72 | 5.37 | 6.04 | 6.81 |
| 36 | 4.77 | 0.21 | 0.49 | 1.66 | 9.84 | 3.06 | 3.60 | 4.16 | 4.77 | 5.41 | 6.09 | 6.86 |
| 37 | 4.81 | 0.21 | 0.51 | 1.66 | 10.09 | 3.08 | 3.64 | 4.20 | 4.81 | 5.46 | 6.14 | 6.91 |
| 38 | 4.85 | 0.21 | 0.52 | 1.66 | 10.34 | 3.11 | 3.67 | 4.23 | 4.85 | 5.50 | 6.19 | 6.95 |
| 39 | 4.89 | 0.21 | 0.53 | 1.66 | 10.60 | 3.14 | 3.70 | 4.27 | 4.89 | 5.55 | 6.23 | 7.00 |
| 40 | 4.93 | 0.21 | 0.54 | 1.66 | 10.85 | 3.16 | 3.73 | 4.31 | 4.93 | 5.59 | 6.28 | 7.05 |
| 41 | 4.97 | 0.21 | 0.56 | 1.66 | 11.10 | 3.19 | 3.76 | 4.34 | 4.97 | 5.64 | 6.33 | 7.10 |
| 42 | 5.01 | 0.20 | 0.57 | 1.66 | 11.35 | 3.21 | 3.80 | 4.38 | 5.01 | 5.68 | 6.37 | 7.14 |
| 43 | 5.05 | 0.20 | 0.58 | 1.66 | 11.61 | 3.24 | 3.83 | 4.42 | 5.05 | 5.72 | 6.42 | 7.19 |
| 44 | 5.09 | 0.20 | 0.59 | 1.66 | 11.86 | 3.26 | 3.86 | 4.45 | 5.09 | 5.76 | 6.46 | 7.23 |
| 45 | 5.13 | 0.20 | 0.61 | 1.66 | 12.11 | 3.29 | 3.89 | 4.49 | 5.13 | 5.81 | 6.51 | 7.28 |
| 46 | 5.17 | 0.20 | 0.62 | 1.66 | 12.36 | 3.31 | 3.92 | 4.52 | 5.17 | 5.85 | 6.55 | 7.32 |
| 47 | 5.21 | 0.20 | 0.63 | 1.66 | 12.62 | 3.34 | 3.95 | 4.56 | 5.21 | 5.89 | 6.59 | 7.37 |
| 48 | 5.25 | 0.20 | 0.65 | 1.66 | 12.87 | 3.36 | 3.98 | 4.59 | 5.25 | 5.93 | 6.64 | 7.41 |
| 49 | 5.29 | 0.20 | 0.66 | 1.66 | 13.12 | 3.39 | 4.01 | 4.63 | 5.29 | 5.97 | 6.68 | 7.46 |
| 50 | 5.32 | 0.20 | 0.67 | 1.66 | 13.37 | 3.41 | 4.04 | 4.66 | 5.32 | 6.01 | 6.72 | 7.50 |
| 51 | 5.36 | 0.20 | 0.68 | 1.66 | 13.63 | 3.44 | 4.07 | 4.70 | 5.36 | 6.05 | 6.76 | 7.54 |
| 52 | 5.40 | 0.20 | 0.70 | 1.66 | 13.88 | 3.46 | 4.10 | 4.73 | 5.40 | 6.09 | 6.80 | 7.58 |
| 53 | 5.44 | 0.20 | 0.71 | 1.66 | 14.13 | 3.48 | 4.13 | 4.77 | 5.44 | 6.13 | 6.84 | 7.62 |
| 54 | 5.47 | 0.20 | 0.72 | 1.66 | 14.38 | 3.51 | 4.16 | 4.80 | 5.47 | 6.17 | 6.89 | 7.66 |
| 55 | 5.51 | 0.20 | 0.73 | 1.66 | 14.64 | 3.53 | 4.19 | 4.83 | 5.51 | 6.21 | 6.93 | 7.70 |
| 56 | 5.55 | 0.20 | 0.75 | 1.66 | 14.89 | 3.55 | 4.22 | 4.86 | 5.55 | 6.25 | 6.97 | 7.74 |
| 57 | 5.58 | 0.20 | 0.76 | 1.66 | 15.14 | 3.58 | 4.24 | 4.90 | 5.58 | 6.29 | 7.00 | 7.78 |
| 58 | 5.62 | 0.20 | 0.77 | 1.66 | 15.39 | 3.60 | 4.27 | 4.93 | 5.62 | 6.33 | 7.04 | 7.82 |
| 59 | 5.65 | 0.20 | 0.78 | 1.66 | 15.65 | 3.62 | 4.30 | 4.96 | 5.65 | 6.36 | 7.08 | 7.86 |
| 60 | 5.69 | 0.20 | 0.80 | 1.66 | 15.90 | 3.64 | 4.33 | 4.99 | 5.69 | 6.40 | 7.12 | 7.90 |
| 61 | 5.72 | 0.20 | 0.81 | 1.66 | 16.15 | 3.67 | 4.36 | 5.03 | 5.72 | 6.44 | 7.16 | 7.94 |
| 62 | 5.76 | 0.19 | 0.82 | 1.66 | 16.40 | 3.69 | 4.38 | 5.06 | 5.76 | 6.48 | 7.20 | 7.97 |
| 63 | 5.79 | 0.19 | 0.84 | 1.66 | 16.66 | 3.71 | 4.41 | 5.09 | 5.79 | 6.51 | 7.23 | 8.01 |
| 64 | 5.83 | 0.19 | 0.85 | 1.66 | 16.91 | 3.73 | 4.44 | 5.12 | 5.83 | 6.55 | 7.27 | 8.05 |
| 65 | 5.86 | 0.19 | 0.86 | 1.66 | 17.16 | 3.75 | 4.46 | 5.15 | 5.86 | 6.58 | 7.31 | 8.08 |
| 66 | 5.89 | 0.19 | 0.87 | 1.66 | 17.41 | 3.77 | 4.49 | 5.18 | 5.89 | 6.62 | 7.34 | 8.12 |
| 67 | 5.93 | 0.19 | 0.89 | 1.66 | 17.67 | 3.79 | 4.52 | 5.21 | 5.93 | 6.65 | 7.38 | 8.15 |
| 68 | 5.96 | 0.19 | 0.90 | 1.66 | 17.92 | 3.82 | 4.54 | 5.24 | 5.96 | 6.69 | 7.41 | 8.19 |
| 69 | 5.99 | 0.19 | 0.91 | 1.66 | 18.17 | 3.84 | 4.57 | 5.27 | 5.99 | 6.72 | 7.45 | 8.22 |
| 70 | 6.03 | 0.19 | 0.92 | 1.66 | 18.42 | 3.86 | 4.60 | 5.30 | 6.03 | 6.76 | 7.48 | 8.26 |
| 71 | 6.06 | 0.19 | 0.94 | 1.66 | 18.68 | 3.88 | 4.62 | 5.33 | 6.06 | 6.79 | 7.52 | 8.29 |
| 72 | 6.09 | 0.19 | 0.95 | 1.66 | 18.93 | 3.90 | 4.65 | 5.36 | 6.09 | 6.82 | 7.55 | 8.33 |
| 73 | 6.12 | 0.19 | 0.96 | 1.66 | 19.18 | 3.92 | 4.67 | 5.39 | 6.12 | 6.86 | 7.58 | 8.36 |
| 74 | 6.15 | 0.19 | 0.97 | 1.66 | 19.43 | 3.94 | 4.70 | 5.42 | 6.15 | 6.89 | 7.62 | 8.39 |
| 75 | 6.19 | 0.19 | 0.99 | 1.66 | 19.69 | 3.96 | 4.73 | 5.45 | 6.19 | 6.92 | 7.65 | 8.42 |
| 76 | 6.22 | 0.19 | 1.00 | 1.66 | 19.94 | 3.98 | 4.75 | 5.48 | 6.22 | 6.96 | 7.68 | 8.46 |
| 77 | 6.25 | 0.19 | 1.01 | 1.66 | 20.19 | 4.00 | 4.78 | 5.51 | 6.25 | 6.99 | 7.72 | 8.49 |
| 78 | 6.28 | 0.19 | 1.03 | 1.66 | 20.44 | 4.02 | 4.80 | 5.54 | 6.28 | 7.02 | 7.75 | 8.52 |
| 79 | 6.31 | 0.19 | 1.04 | 1.66 | 20.70 | 4.04 | 4.83 | 5.57 | 6.31 | 7.05 | 7.78 | 8.55 |
| 80 | 6.34 | 0.19 | 1.05 | 1.66 | 20.95 | 4.06 | 4.85 | 5.59 | 6.34 | 7.09 | 7.81 | 8.59 |
| 81 | 6.37 | 0.19 | 1.06 | 1.66 | 21.20 | 4.08 | 4.88 | 5.62 | 6.37 | 7.12 | 7.85 | 8.62 |
| 82 | 6.40 | 0.19 | 1.08 | 1.66 | 21.45 | 4.10 | 4.90 | 5.65 | 6.40 | 7.15 | 7.88 | 8.65 |
| 83 | 6.43 | 0.18 | 1.09 | 1.66 | 21.71 | 4.12 | 4.93 | 5.68 | 6.43 | 7.18 | 7.91 | 8.68 |
| 84 | 6.47 | 0.18 | 1.10 | 1.66 | 21.96 | 4.14 | 4.95 | 5.71 | 6.47 | 7.21 | 7.94 | 8.71 |
| 85 | 6.50 | 0.18 | 1.11 | 1.66 | 22.21 | 4.16 | 4.98 | 5.74 | 6.50 | 7.24 | 7.98 | 8.74 |
| 86 | 6.53 | 0.18 | 1.13 | 1.66 | 22.46 | 4.17 | 5.00 | 5.76 | 6.53 | 7.28 | 8.01 | 8.77 |
| 87 | 6.56 | 0.18 | 1.14 | 1.66 | 22.72 | 4.19 | 5.03 | 5.79 | 6.56 | 7.31 | 8.04 | 8.80 |
| 88 | 6.59 | 0.18 | 1.15 | 1.66 | 22.97 | 4.21 | 5.05 | 5.82 | 6.59 | 7.34 | 8.07 | 8.83 |
| 89 | 6.62 | 0.18 | 1.16 | 1.66 | 23.22 | 4.23 | 5.08 | 5.85 | 6.62 | 7.37 | 8.10 | 8.87 |
| 90 | 6.65 | 0.18 | 1.18 | 1.66 | 23.47 | 4.25 | 5.10 | 5.88 | 6.65 | 7.40 | 8.13 | 8.90 |
| 91 | 6.68 | 0.18 | 1.19 | 1.66 | 23.73 | 4.27 | 5.13 | 5.91 | 6.68 | 7.43 | 8.16 | 8.93 |
| 92 | 6.71 | 0.18 | 1.20 | 1.66 | 23.98 | 4.29 | 5.15 | 5.93 | 6.71 | 7.46 | 8.20 | 8.96 |
| 93 | 6.74 | 0.18 | 1.21 | 1.66 | 24.23 | 4.31 | 5.18 | 5.96 | 6.74 | 7.50 | 8.23 | 8.99 |
| 94 | 6.77 | 0.18 | 1.23 | 1.66 | 24.48 | 4.33 | 5.20 | 5.99 | 6.77 | 7.53 | 8.26 | 9.02 |
| 95 | 6.80 | 0.18 | 1.24 | 1.66 | 24.74 | 4.35 | 5.22 | 6.02 | 6.80 | 7.56 | 8.29 | 9.05 |
| 96 | 6.83 | 0.18 | 1.25 | 1.66 | 24.99 | 4.37 | 5.25 | 6.05 | 6.83 | 7.59 | 8.32 | 9.08 |
| 97 | 6.86 | 0.18 | 1.27 | 1.66 | 25.24 | 4.39 | 5.27 | 6.08 | 6.86 | 7.62 | 8.35 | 9.11 |
| 98 | 6.89 | 0.18 | 1.28 | 1.66 | 25.49 | 4.41 | 5.30 | 6.10 | 6.89 | 7.65 | 8.38 | 9.14 |
| 99 | 6.92 | 0.18 | 1.29 | 1.66 | 25.75 | 4.43 | 5.32 | 6.13 | 6.92 | 7.68 | 8.41 | 9.17 |
| 100 | 6.95 | 0.18 | 1.30 | 1.66 | 26.00 | 4.45 | 5.35 | 6.16 | 6.95 | 7.71 | 8.45 | 9.20 |

**x**: is the age of child in weeks; C: Percentile

| (Female, height) (MICS vs NUTR) | (Male, height) (MICS vs NUTR) |
| --- | --- |
| (Female, weight) (MICS vs NUTR) | (Male, weight) (MICS vs NUTR) |

Figure S1 Comparison of 3^rd^, 50^th^ and 97^th^ percentiles (W/A and H/A) of this study with other study based on national nutritional survey data
